# Proteomic profiling of single extracellular vesicles reveals association of CD31^+^ EV subpopulation with immune dysregulation in people living with HIV

**DOI:** 10.3389/fimmu.2026.1871641

**Published:** 2026-07-07

**Authors:** Ning Ding, Jiewei Sun, Aiwei Zhu, Jiajun Li, Jin Sun, Hongxia Yan, Yuanyuan Ma, Xiangchuan He, Tong Zhang, Qi Yao, Bin Su

**Affiliations:** 1 Beijing Key Laboratory for HIV/AIDS Research, Sino-French Joint Laboratory for HIV/AIDS Research, Clinical and Research Center for Infectious Diseases, Beijing Youan Hospital, Capital Medical University, Beijing, China; 2 The Scientific and Technological Achievement Transformation Center, Beijing Youan Hospital, Capital Medical University, Beijing, China; 3 Central Laboratory, Beijing Youan Hospital, Capital Medical University, Beijing, China

**Keywords:** CD31 (PECAM1), extracellular vesicle subpopulation, HIV, immune dysregulation, proteomic profiling

## Abstract

**Background:**

HIV remains globally prevalent, and the long-term complications experienced by people living with HIV (PLWH) have increased interest in the functional heterogeneity of extracellular vesicle (EV) subpopulations. Defining these EV subsets may help clarify HIV-associated immune dysregulation and inform future diagnostic and therapeutic strategies.

**Methods:**

Plasma samples were obtained from three PLWH (HIV^+^ group) and three healthy controls (HC group). Single-EV surface membrane proteins were profiled using the proximity barcoding assay (PBA), and EV subpopulations were identified by FlowSOM clustering with t-SNE visualization. Functional enrichment analysis, protein-protein interaction (PPI) network analysis, and database-based annotation were used to infer putative subpopulation functions and cellular origins. Differentially expressed EV proteins (DEPs) were screened using limma, and ligand-receptor interaction networks were constructed with CellPhoneDB and cross-referenced with publicly available single-cell RNA sequencing data.

**Results:**

HIV infection did not appear to alter overall EV abundance but was associated with selective remodeling of EV heterogeneity and expansion of distinct EV subpopulations. CD31^+^ EVs (cluster 2), inferred to be endothelial-derived, were markedly enriched in PLWH. Ligand-receptor analysis suggested that CD31^+^ EVs may communicate with CD16^+^ monocytes through F11R-ITGAL/ITGB2 and with plasmablasts through CD31-CD38 interactions; these interactions were associated with inflammatory, leukocyte transendothelial migration, and metabolic pathways. In addition, HIV-enriched B2M^+^ EVs (clusters 3) and MUC16^+^ EVs (clusters 9) showed predicted interactions with CD4^+^ T cells and with CD8^+^ effector memory T and NK cells, respectively, suggesting potential effects on reservoir-related and cytotoxicity-related programs.

**Conclusions:**

These exploratory findings suggest that EV heterogeneity may encode cell-type-specific immune regulatory information in HIV infection and highlight CD31^+^ EVs as a candidate EV subset associated with HIV-related immune dysregulation. Larger cohorts and functional validation are required before these EV populations can be considered therapeutic targets for HIV cure strategies.

## Introduction

Human immunodeficiency virus (HIV) infection remains a major global public health challenge. According to the latest UNAIDS report, approximately 40 million people were living with HIV (PLWH) worldwide by the end of 2024 (1). The widespread use of combination antiretroviral therapy (ART) has substantially improved the prognosis of PLWH, transforming HIV infection from a fatal illness into a chronic, manageable condition (2). Nevertheless, although effective ART suppresses viral replication and reduces AIDS-related mortality, it does not eliminate latent viral reservoirs, which are primarily harbored in CD4^+^ central memory T cells, nor does it fully resolve chronic immune activation and immune dysfunction (3, 4). Even during sustained viral suppression, 10% to 40% of PLWH show inadequate CD4^+^ T cell recovery, commonly referred to as an immunological non-responder (INR) (5). This condition leaves patients vulnerable to opportunistic infections, immune senescence, and non-AIDS comorbidities, including cardiovascular disease, neurocognitive impairment, and malignancies (6). Proteomic studies have further shown that the plasma proteome undergoes dynamic remodeling during hyperacute HIV-1 infection, and that early perturbations in inflammatory and immune-regulatory proteins may contribute to the molecular foundation basis of chronic immune activation (7).

These pathological outcomes are closely associated with persistent inflammation, which is driven by residual viral antigens, microbial translocation, and dysregulated intercellular communication within the HIV-infected microenvironment (8, 9). Increasing evidence indicates that non-classical communication pathways beyond direct cell-cell contact, particularly EV-mediated signaling, may participate in sustaining chronic immune activation (10). Therefore, defining the molecular mechanisms that link chronic immune activation with reservoir persistence remains an important barrier in HIV cure research. EVs are lipid-bilayer-enclosed nanoparticles secreted by almost all cell types and can transfer proteins, lipids, and nucleic acids to recipient cells through ligand-receptor interactions. Prior studies suggest that HIV-infected cells may use EVs to modulate immune signaling, support viral evasion, and contribute to reservoir maintenance, for example, by transferring viral proteins such as Nef and Tat or host-derived immune regulators to target cells (11–13). Plasma neuronal exosomes have also been reported as biomarkers of cognitive impairment in HIV infection and Alzheimer’s disease (14).

Circulating EVs arise from diverse cell types and carry cell-type-specific molecular cargo that reflects the physiological state of their parental cell (15). However, most EVs studies have relied on bulk-level analyses, which average signals across heterogeneous vesicle populations and may obscure the functional contribution of disease-associated EV subsets (12, 16). This limitation is especially relevant in HIV infection, where subtle but biologically meaningful EV subpopulations may contribute to immune dysregulation. Recent single-vesicle technologies, including the proximity barcoding assay (PBA), have begun to address this limitation by enabling the multiplexed detection of hundreds of membrane proteins on individual EVs (17). Nevertheless, the single-vesicle features of HIV-associated plasma EV remodeling remain incompletely characterized, and the extent to which cell-type-specific EV subsets may participate in targeted immune regulation, chronic inflammation, and reservoir persistence has not been systematically evaluated.

Endothelial dysfunction is a recognized feature of chronic HIV infection and contributes to vascular inflammation and non-AIDS comorbidities (18, 19). Endothelial cells express adhesion molecules such as platelet endothelial cell adhesion molecule-1 (PECAM1/CD31), which regulates leukocyte adhesion, transmigration, and vascular homeostasis (20). CD31 is involved in maintaining endothelial barrier homeostasis and has also been implicated in CD4^+^ T cell survival (21), suggesting that CD31 carried by endothelial-derived EVs could influence CD4^+^ T cells through transcellular delivery in HIV infection. Endothelial cell-derived EVs have been implicated in inflammatory and vascular diseases, where they can modulate immune cell behavior through specific ligand-receptor interactions (22, 23). Because the vascular endothelium is an important component of the systemic immune microenvironment, endothelial dysfunction may contribute to systemic inflammation and tissue injury in chronic disease (24). These observations support the potential relevance of endothelial-derived EVs in HIV immunopathology. However, whether endothelial-derived EV subpopulations contribute to HIV-associated immune dysregulation through communication with key immune cells remains largely unexplored.

In the present study, we integrated EV cluster-based PBA with single-cell RNA sequencing (scRNA-seq) analysis to characterize the heterogeneity, inferred cellular origins, and potential immunomodulatory functions of HIV-associated EVs. We hypothesized that HIV infection remodels EV subpopulation composition rather than bulk EV abundance, and that distinct EV subpopulations may participate in cell-type-specific immune regulation related to viral persistence. We further aimed to connect predicted EV ligand-receptor interactions with downstream transcriptomic differences in immune cells. This exploratory analysis proposes a single-vesicle framework for studying EV-mediated immunomodulation in HIV infection, immune dysregulation, and reservoir maintenance.

## Materials and methods

### Study design and sample

This retrospective study used archived plasma specimens from three people living with HIV (HIV^+^ group) and three age-matched healthy controls (HC group). Basic demographic and available clinical information, including age, sex, and HIV diagnosis status, were extracted from existing medical records (**Table 1**).

**Table 1:** Demographic characteristics and clinical features of study participants.

Characteristics	HIV+ group (n = 3)	HC group (n = 3)	p value
Gender, n (%)			
Male	3 (100%)	3 (100%)	1
Female	NA	NA	
Age, years	23 ± 2.5	29 ± 5.7	0.19
ART status, n (%)			
Pre-ART	2 (66.6%)	NA	NA
Post-ART	1 (33.3%)	NA	NA
HIV viral load (log10 copies/mL)	3.32 ± 1.52	NA	NA
CD4+ T-cell count, cells/μL	294.5 ± 105	NA	NA
Comorbidities, n (%)			
Syphilis	NA	NA	NA
Diabetes	NA	NA	NA
Hypertension	NA	NA	NA
HBV	1 (33.3%)	NA	NA
PCP	NA	NA	NA

ART, antiretroviral therapy; HBV, hepatitis B virus; PCP, Pneumocystis pneumonia; NA, not applicable.

The inclusion criteria were as follows: (I) PLWH were diagnosed with HIV infection according to the Chinese guidelines for the diagnosis and treatment of HIV infection/AIDS; (II) all PLWH had well-documented clinical records, including HIV seropositivity and basic immunological profiles; (III) healthy controls had no history of HIV infection, chronic infectious disease, systemic disease, or autoimmune disorder; (IV) all participants were aged 18–65 years; and (V) archived plasma samples had sufficient quality and volume for EV proteomic analysis.

### Sample collection

All specimens were collected at the Clinical and Research Center for Infectious Diseases and preserved at −80 °C under standardized biobanking conditions. Study participants provided venous blood samples following an overnight fast. In brief, 10 mL of venous blood were transported to the laboratory and processed within 2 h after venipuncture. Plasma was separated by centrifugation at 1,500 rpm for 10 min at room temperature, with acceleration and deceleration parameters at level 4. After centrifugation, the plasma supernatant was aspirated without disturbing the buffy coat or cellular fraction. Plasma was centrifuged at 2000 g for 10 min, 12000 g for 30 min to remove cell debris, and the resulting supernatant was harvested for PBA testing. The detailed flowchart is presented in **Supplementary Figure 1**.

### Single-vesicle membrane proteomic profiling using PBA

Membrane proteins on individual extracellular vesicles were profiled using a PBA with the Vesicode^®^ Surface550 panel (Secretech, Shanghai, China) (17). Briefly, the PBA platform uses approximately 550 oligonucleotide-labeled antibodies to simultaneously detect EV surface proteins. Each antibody is conjugated to a unique DNA barcode containing a protein tag that identifies the protein type and a molecular tag that supports duplicate-count correction. Platelet-poor plasma samples were applied to surfaces coated with cholera toxin subunit B (CTB), enabling vesicle capture through GM1 ganglioside binding. Antibody–oligonucleotide conjugates bound to proteins on the same EV were assigned a shared EV-specific barcode, allowing reconstruction of protein co-localization at the single-vesicle level. DNA constructs encoding EV tag, protein tag, and molecule tag information were generated through proximity extension and ligation reactions, followed by PCR amplification for sequencing library preparation. Sequencing was performed in paired-end 150 bp (PE150) mode using either the DNBSEQ-T7 platform (MGI, Shenzhen, China) or the NovaSeq S4 (Illumina, USA). Raw sequencing data were converted from BCL format to FASTQ files using MegaBolt for MGI platform or bcl2fastq for Illumina platform.

### Single-EV clustering and subpopulation characterization

Raw sequencing reads were processed with the EVisualizer^®^ decoding package (version 1.1.6, Secretech, Shenzhen, China) to generate EV-level protein expression matrices, including EV ID–protein expression datasets, protein-combination datasets, and EV subpopulation annotations. Total protein expression across EVs was summarized and normalized using the trimmed mean of M-values (TMM) method to correct for differences in library-size. Protein co-expression patterns, defined as the detection of two or more proteins on the same EV, were summarized as protein-combination datasets and normalized as counts per million (CPM). To account for EV heterogeneity, vesicles were stratified according to the number of protein cargos per vesicle into four subtypes: comb1 (one protein), comb2 (two proteins), comb3 (three proteins), and comb4More (four or more proteins). Differential expression analysis of EV membrane proteins between the HIV^+^ group and HC group was performed using the limma package in R. Raw protein count data were processed with voom transformation and quantile normalization, followed by linear model fitting (lmFit) and empirical Bayes moderation (eBayes). Proteins were considered significantly differentially expressed under the criteria of a nominal *p*-value < 0.05 and an absolute |log_2_ fold change (FC)| ≥ 1. Volcano plots, hierarchical clustering heatmaps, and box plots were generated to visualize differential expression patterns and sample distribution. Unsupervised clustering of single EVs was performed using the FlowSOM algorithm based on membrane protein expression profiles of comb2, comb3, and comb4More vesicles. Dimensionality reduction was performed using t-SNE and UMAP. EV subpopulations were defined by metaclustering, and cluster-specific marker proteins were identified according to enrichment within each cluster. The relative abundance of each EV subpopulation was calculated as the proportion of EVs assigned to each cluster relative to the total EV population in each sample. Pearson correlation matrices of average protein expression across EV clusters were calculated and visualized as heatmaps via the R pheatmap package. Based on the two key HIV-upregulated markers B2M and PECAM1, an HIV risk score (0–10) was defined and stratified by group (HIV^+^ group versus HC group), and facet t-SNE and violin plots were generated using R ggplot2 from the tidyverse and statistical significance annotated by ggsignif. Hierarchical clustering based on inter-cluster Pearson correlation was used to construct an EV cluster tree, and cluster relative abundances were displayed as heatmaps using pheatmap. EV pseudotime trajectories were inferred using the R princurve package on t-SNE coordinates, and pseudotime-colored subpopulations, trajectory lines, and cluster labels were visualized using ggplot2 and ggrepel.

### Cellular origin inference and functional enrichment analysis

To infer the potential cellular origins of EV clusters, cluster marker proteins were compared with known cell-type-specific markers in the CellMarker database. The dplyr package was used to grouped and summarized marker data by EV cluster and cell type, quantify marker counts, and calculated the relative contribution of each cell type to individual clusters. Functional annotation of cluster marker proteins and differentially expressed proteins (DEPs) was performed using the DisGeNET database (25) and KOBAS 3.0 to identify disease associations.

### Integration with single-cell RNA-seq data

Public single-cell RNA sequencing data from 14 peripheral blood samples (GEO accession: GSE220790) were analyzed to define immune cell subsets and receptor expression patterns. Raw sequencing data were processed and analyzed in R software using the Seurat package together with auxiliary packages, including dplyr, ggplot2, patchwork, and tidyr. UMAP was used for dimensionality reduction and visualization of cell subpopulations. Cell types were annotated by calculating the average expression scores for canonical immune-cell marker gene sets, and cells with low signature scores were classified as unknown.

### Ligand–receptor interaction and communication-strength analysis

Ligand-receptor pairs were predicted using the CellPhoneDB database (v5.0.0) with EV membrane proteins annotated as potential ligands. Directed circular interaction networks for key EV subpopulations, including clusters 2, 3, and 9, were constructed and visualized in R using the igraph, ggraph, ggplot2, and patchwork packages. Ligand-receptor interaction analysis was performed in R using the tidyverse, ggplot2, and scales packages. Communication scores were calculated as the product of EV ligand expression and the average expression of the corresponding receptors in each immune cell types. A permutation test with 1,000 iterations was used to assess statistical significance, and P-values were adjusted for multiple testing using the false discovery rate (FDR). For each significant ligand-receptor pair identified in the intercellular communication analysis, module scores for the corresponding receptor gene sets were calculated in each target immune cell type using the AddModuleScore function in Seurat.

### Transcriptomic reprogramming in receptor-stratified immune cell subsets

Within each corresponding immune cell type, receptor module scores were calculated, and cells were stratified into receptor-high and receptor-low groups using the median module score as the cutoff. Differentially expressed genes (DEGs) between receptor-high and receptor-low cells were identified using the FindMarkers function with the following thresholds of |log_2_ FC| > 0.5 and *p*-value < 0.01. Volcano plots were generated using ggplot2 to visualize DEGs. Functional interpretation of DEGs was performed using KOBAS and Cytoscape 3.10.4.

### Statistics

The statistical analysis of the PBA data is detailed in the Data Processing and Analysis section. All the other statistical analyses were conducted using GraphPad Prism (v8). Continuous variables are presented as the mean ± SEM. Student’s t-test and the Wilcoxon test were applied for comparisons involving only two groups, and were two-sided unless otherwise specified. In general, a *p*-value less than 0.05 indicated statistically significant.

## Results

### HIV infection reshapes EV proteomic profiles

To characterize circulating EV membrane proteins in HIV infection, plasma samples from the HC group and HIV^+^ group were profiled using the PBA. A total of 543 membrane proteins were identified, covering a broad spectrum of surface molecules, including adhesion molecules, immune receptors, signaling proteins, and transporters. Gene Ontology (GO) enrichment analysis showed that these proteins were significantly enriched in biological processes related to immune regulation and cell-cell communication (**Supplementary Figure 2**).

**Figure 1:**
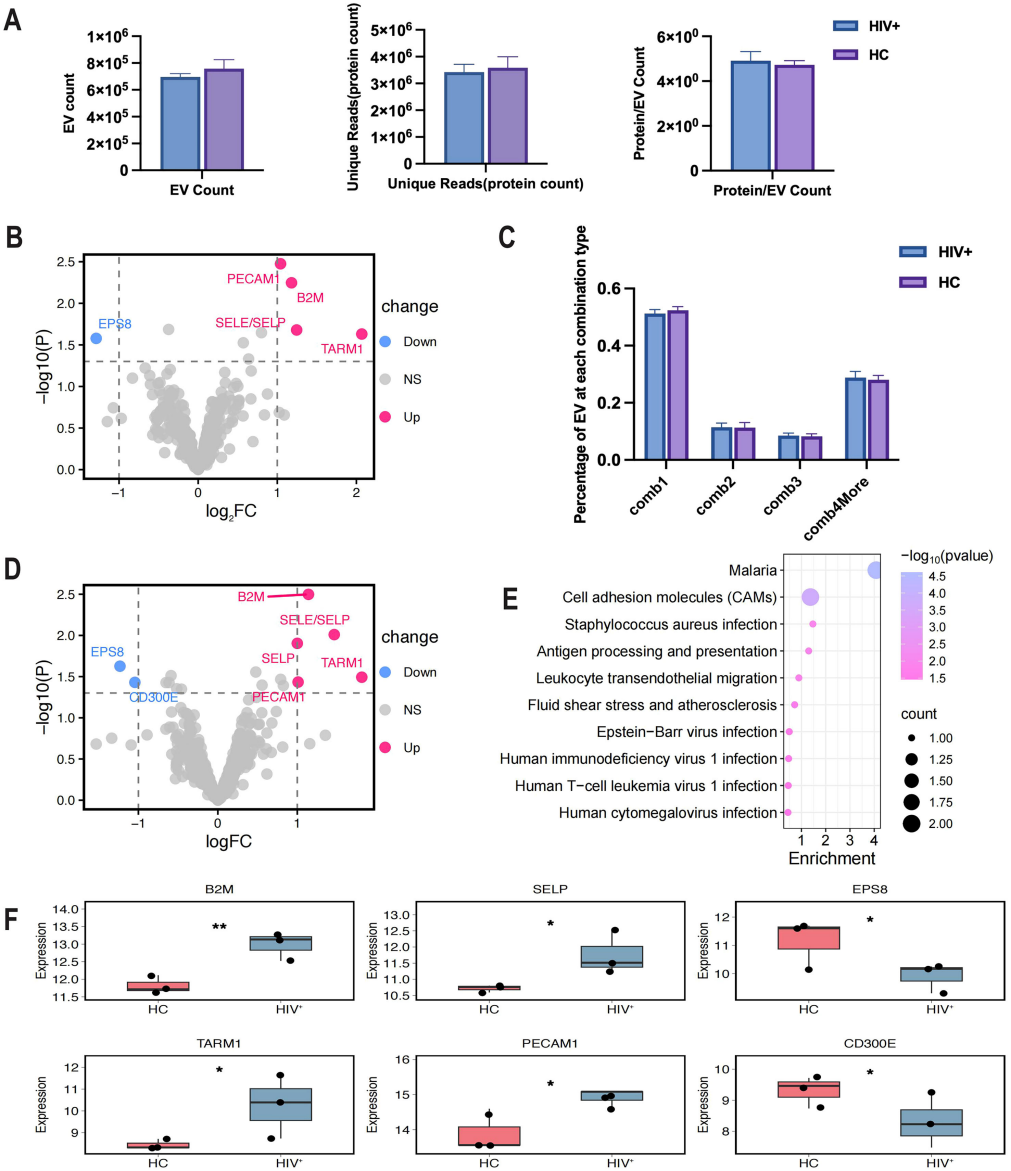
Characterization of EV protein profiles and differential expression analysis. **(A)** Quantification of EV counts, total protein counts, and protein/EV ratios in HC group and HIV^+^ group. **(B)** Volcano plot of differential protein expression in the total EV dataset. Based on *p* < 0.05 and |log_2_FC|≥ 1, four proteins were significantly upregulated, and one protein was significantly downregulated in HIV^+^ group compared with HC group. **(C)** Proportion distribution of distinct EV protein combinations (comb1–comb4More) across all samples prior to subpopulation purification. comb1: EV carrying exactly one detected protein; comb2: EV carrying exactly two detected proteins; comb3: EV carrying exactly three detected proteins; comb4More: EV carrying four or more detected proteins. **(D)** Volcano plot of differential protein expression in the subsets, including comb 2, comb 3, and comb4More. Based on *p* < 0.05 and |log_2_FC|≥ 1, five proteins were significantly upregulated and two proteins were significantly downregulated in HIV^+^ group compared with HC group. **(E)** Box plots showing the expression differences of core differentially expressed proteins between the HC group and HIV^+^ group (*p < 0.05, **p < 0.01). **(F)** Bubble plot of significantly enriched pathways for differentially expressed proteins in the comb 2, comb 3, and comb4More (adjusted *p* < 0.05).

Baseline EV characteristics did not differ significantly between the HC group and HIV^+^ group in EV count, total protein count, or protein-to-EV ratio (**Figure 1A**), suggesting that HIV infection did not markedly change overall plasma EV abundance or protein-loading capacity in this cohort. Differential expression analysis of the full EV proteome identified multiple dysregulated proteins (**Figure 1B**): PECAM1, B2M, SELP/SELE, and TARM1 were upregulated in HIV^+^ group, whereas EPS8 was downregulated. To incorporate EV heterogeneity, we compared comb1, comb2, comb3, and comb4More vesicles across groups (**Figure 1C**). After excluding the predominant single-protein comb1 subset, we repeated differential analysis in the combined multivalent EV population (comb2, comb3, and comb4More), which again supported the core differential proteins (**Figure 1D**), with corresponding expression patterns shown in box plots (**Figure 1E**). KEGG pathway enrichment indicated that these dysregulated proteins were strongly enriched in immune-related processes, including cell adhesion molecules (CAMs), antigen processing and presentation, leukocyte transendothelial migration, as well as HIV-1 infection, and other viral, bacterial, and parasitic infection pathways (**Figure 1F**). These results suggest that HIV-associated EV alterations may not be primarily reflected at the bulk EV level but may instead arise from changes in EV subpopulation composition, motivating further analysis at single-vesicle resolution.

### Single-vesicle analysis identifies expansion of HIV-associated EV subpopulations

**Figure 2:**
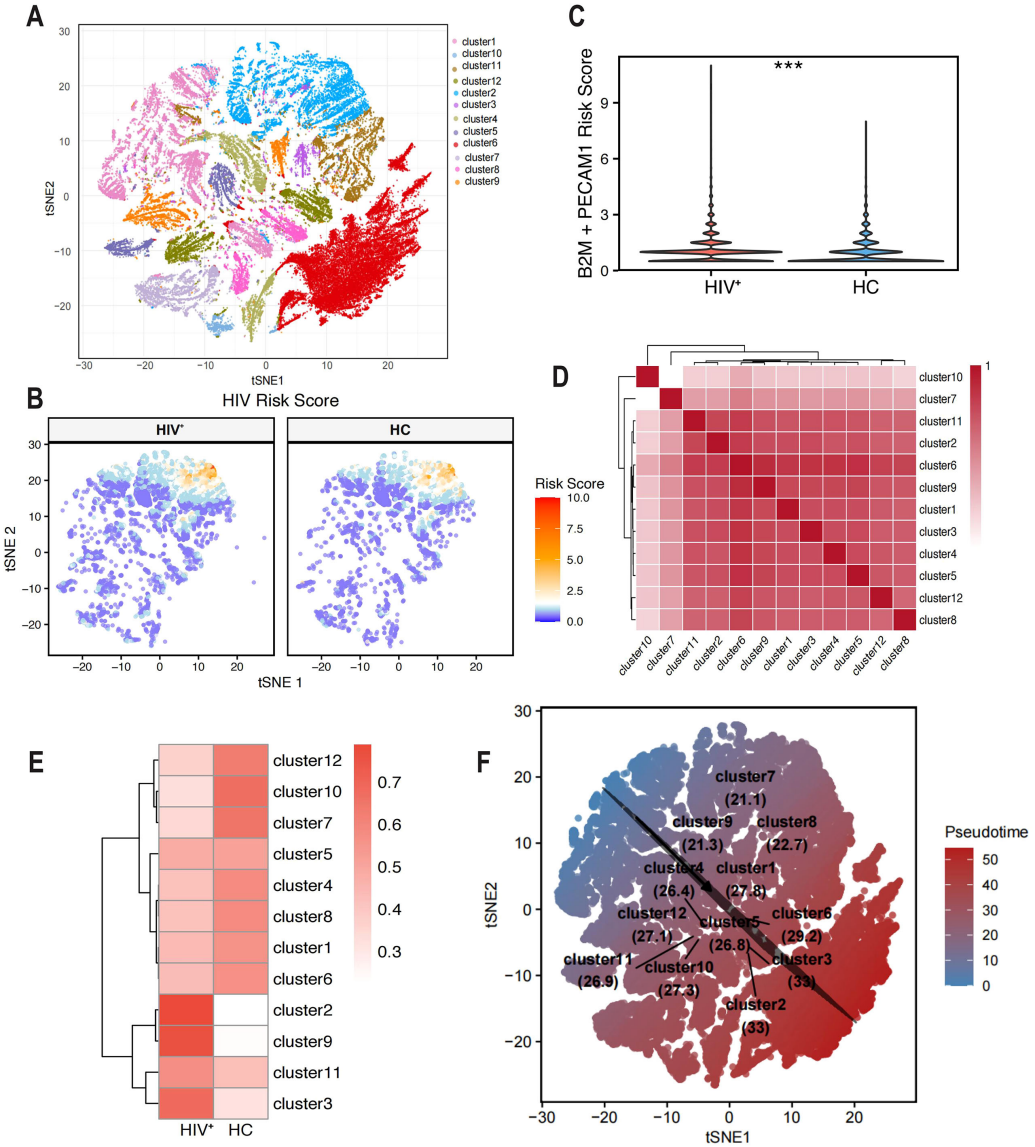
Single-EV profiling identifies HIV-associated EV features and reveals EV subpopulation heterogeneity. **(A)** tSNE visualization of 12 distinct EV clusters identified by unsupervised clustering based on global protein expression profiles. **(B)** The tSNE plots showing the distribution of HIV risk scores (derived from B2M and PECAM1 co-expression) in EVs from HIV^+^ group **(left)** and HC group (right). **(C)** Violin plot comparing the HIV risk scores between HIV^+^ group and HC group (***p < 0.001, unpaired t-test), confirming significantly higher risk scores in HIV infection. **(D)** Correlation heatmap of the 12 EV clusters. **(E)** Cluster phylogenetic tree and group abundance heatmap. Hierarchical clustering tree reflecting the similarity of protein expression profiles among 12 EV clusters. **(F)** Pseudotime trajectory of EV subpopulations on t-SNE space. EVs are colored by pseudotime (blue = early stage, red = late stage) with a principal curve indicating inferred trajectory. Cluster labels include mean pseudotime values, delineating early (21.1–22.7), intermediate (26.4–27.8), and late (29.2–33.0) differentiation stages.

**Figure 3:**
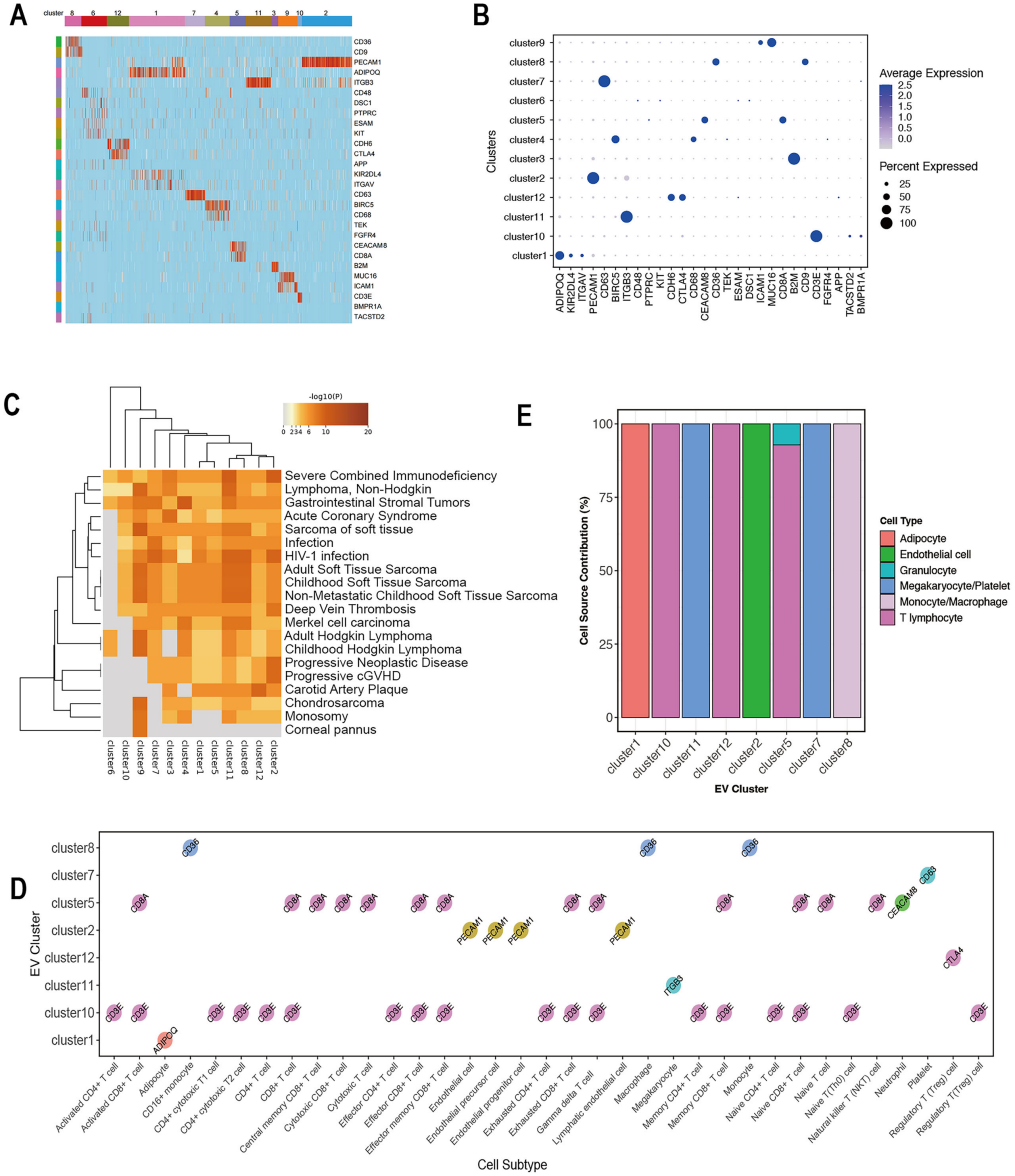
Molecular and predicted cellular origin of EV subsets. **(A)** Heatmap of marker proteins across EV clusters, colored by log10 (*p*-value) enrichment score. **(B)** Dotplot of highly expressed proteins in each EV cluster, with color indicating average expression and size representing the percentage of cells expressing the marker. **(C)** Disease enrichment heatmap of the top 10 expressed proteins per cluster, analyzed using the DisGeNET database. **(D)** Bubble plot showing predicted cell-type associations at the subtype level, with bubble size scaled by marker count and color by major cell type. **(E)** Stacked bar plot summarizing the percentage contribution of each major cell type to the cellular origin of each EV cluster.

To define EV diversity at the single-vesicle resolution, we performed unsupervised clustering based on single-EV protein expression, identifying 12 distinct EV subpopulations (clusters 1-12) across all samples (**Figure 2A**; **Supplementary Table 1**). The relative abundance of the 12 clusters was quantified in each sample in HIV^+^ group and HC group (**Supplementary Figure 3A**), and UMAP visualization confirmed that these clusters were consistently detected across all six samples (**Supplementary Figure 3B**). Based on the two HIV-upregulated markers B2M and PECAM1, we constructed a single-EV-level HIV risk score and found that high-risk-score EVs were substantially more abundant in the HIV^+^ group than in the HC group (**Figures 3B, C**). The 12 clusters showed strong positive correlations across subpopulations, indicating overall similarity in EV protein expression (**Figure 2D**). Hierarchical clustering suggested that clusters 2 and 9, as well as clusters 3 and 11, were closer in inter-cluster distance and associated with HIV infection status, whereas other clusters were predominantly observed in HC group (**Figure 2E**). Pseudotime trajectory analysis further suggested a potential progression from early, less differentiated EV states to later, more mature states (**Figure 2F**). Early low-pseudotime subpopulations included clusters 7, 8, and 9; intermediate transitional subpopulations included clusters 4, 5, 6, 10, 11, and 12; and high-pseudotime subpopulations included clusters 2 and 3, which were positioned sequentially along the inferred trajectory. Together with the enrichment patterns in **Figure 2E**, these results suggest that late-stage clusters 2 and 3 and early-stage cluster 9 represent HIV-associated EV subpopulations potentially involved in immune dysregulation.

### CD31^+^ EVs (cluster 2) represent a dominant HIV-associated subpopulation

To characterize the molecular features and cellular origins of the 12 EV subclusters, we systematically analyzed marker protein expression, disease enrichment, and cell-type distribution across all clusters. Marker-protein expression profiles across EV clusters were visualized using enrichment scores (**Figure 3A**). Each subpopulation was named according to its signature high-expression and frequency protein, such as CD31^+^ EVs for cluster 2, B2M^+^ EVs for cluster 3, and MUC16^+^ EVs for cluster 9 (**Figure 3B**; **Supplementary Table 2**). The disease-enrichment heatmap of the top 10 expressed proteins per cluster indicated that the protein signatures of specific EV clusters were strongly associated with disease categories including HIV-1 infection, severe combined immunodeficiency, and other immune-related diseases (**Figure 3C**). Notably, clusters enriched in the HIV^+^ group, particularly CD31^+^ EVs, B2M^+^ EVs, and MUC16^+^ EVs, were linked to inflammatory and infectious disease processes. To infer the cellular origins of these EV subpopulations, cell-type mapping was performed at both the subtype and major cell type levels. CD31^+^ EVs showed strong marker enrichment for vascular endothelial cells, whereas CEACAM^+^ CD8A^+^ EVs (cluster 5), CD3E^+^ EVs (cluster 10), and CTLA4^+^ CDH6^+^ EVs (cluster 12) were mainly associated with T lymphocytes (**Figures 3D, E**). These findings suggest that endothelial-derived CD31^+^ EVs constitute the dominant HIV-associated EV subpopulation in this dataset.

### EV subpopulations exhibit distinct functions and ligand-receptor interaction specificities

**Figure 4:**
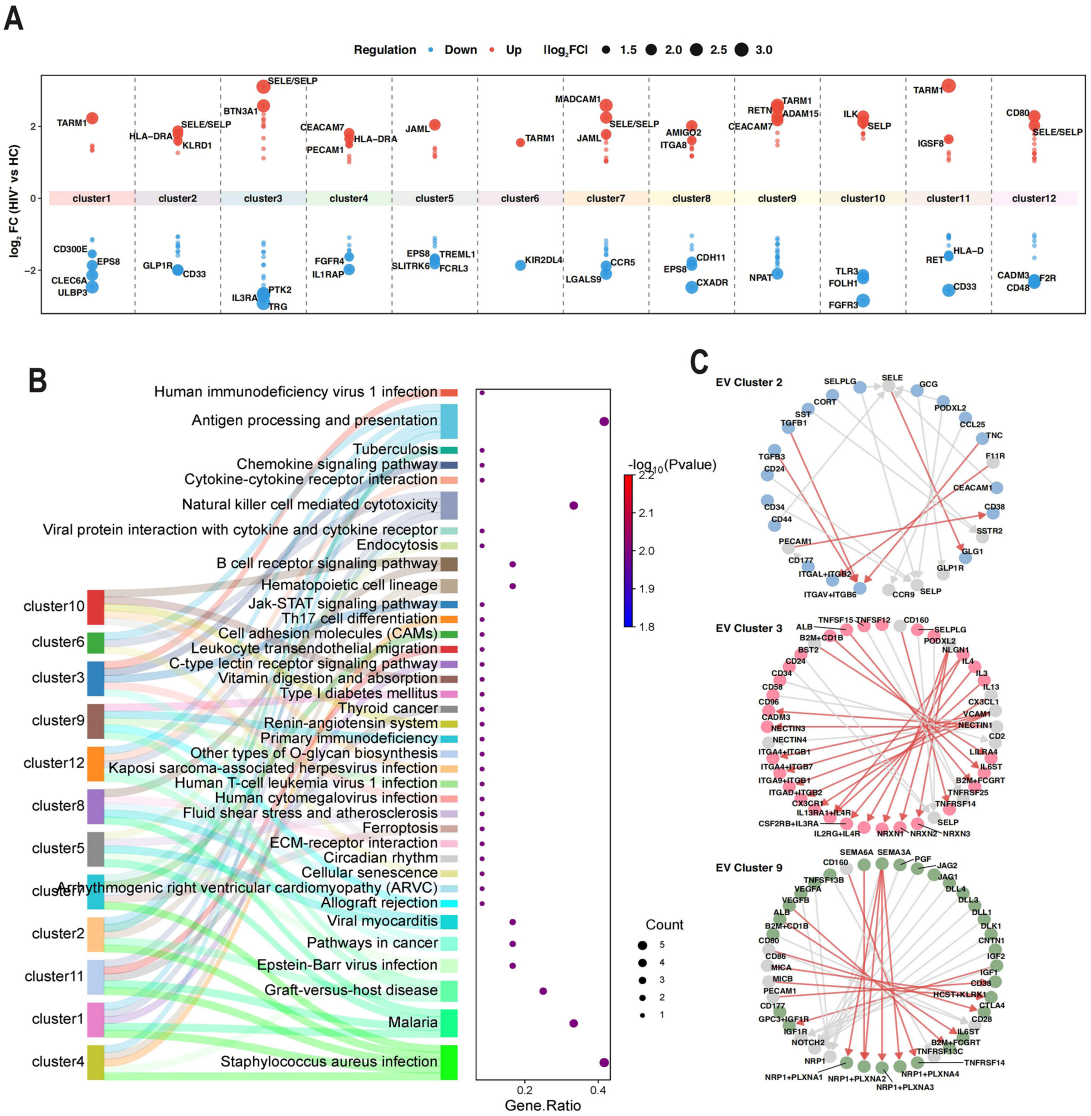
Cluster-specific differential proteins, functional enrichment, and predicted ligand-receptor interaction networks in HIV infection. **(A)** Cluster-specific differential protein expression profile. A dot plot showing log_2_FC of proteins between HIV^+^ and HC group across 12 exosome clusters. **(B)** Functional pathway enrichment analysis. A chord plot linking each EV cluster to its top 5 significantly enriched biological pathways (37 unique pathways in total). **(C)** Circular networks illustrating predicted ligand-receptor interactions involving EV cluster 2 (blue nodes), EV cluster 3 (red nodes), and EV cluster 9 (green nodes). Gray nodes represent EV-associated ligands, whereas colored nodes represent corresponding receptors identified from the scRNA-seq dataset. Lines indicate computationally inferred ligand-receptor pairs.

Cluster-specific differential protein expression between the HIV^+^ group and HC group was first assessed for each EV subpopulation (**Figure 4A**). Functional pathway enrichment analysis of differentially expressed proteins was used to annotate the biological features of each EV cluster. The top five significantly enriched biological pathways for each cluster, comprising 37 unique pathways in total, revealed marked functional heterogeneity across EV subpopulations (**Figure 4B**). Key HIV-associated clusters, including CD31^+^ EVs (cluster 2), B2M^+^ EVs (cluster 3), and MUC16^+^ EVs (cluster 9), were predominantly enriched in immune-related and viral infection pathways, such as human immunodeficiency virus 1 infection, antigen processing and presentation, cell adhesion molecules (CAMs), chemokine signaling pathway, and leukocyte trans endothelial migration.

EVs may participate in intercellular communication through interactions between EV surface proteins and receptors expressed on recipient cells. Ligand-receptor analysis suggested that EV surface proteins from CD31^+^ EVs (cluster 2), B2M^+^ EVs (cluster 3), and MUC16^+^ EVs (cluster 9) could communicate with immune cells through distinct interaction modules (**Figure 4C**; **Supplementary Table 3**). In CD31^+^ EVs, EV-derived CD31 paired with CD38 were EVs-derived F11R paired with ITGAL/ITGB2 were prominent. B2M^+^ EVs were mainly characterized by integrin-related ligands, including EV-derived B2M and CD1B paired with IL6ST, whereas MUC16^+^ EVs carried ligands such as CD80, CD160, and MICA/MICB. Collectively, these results indicate that distinct EV subpopulations may engage recipient cells through specific ligand-receptor pairs repertoires.

### Cluster 2 EVs preferentially target CD16^+^ monocytes

**Figure 5:**
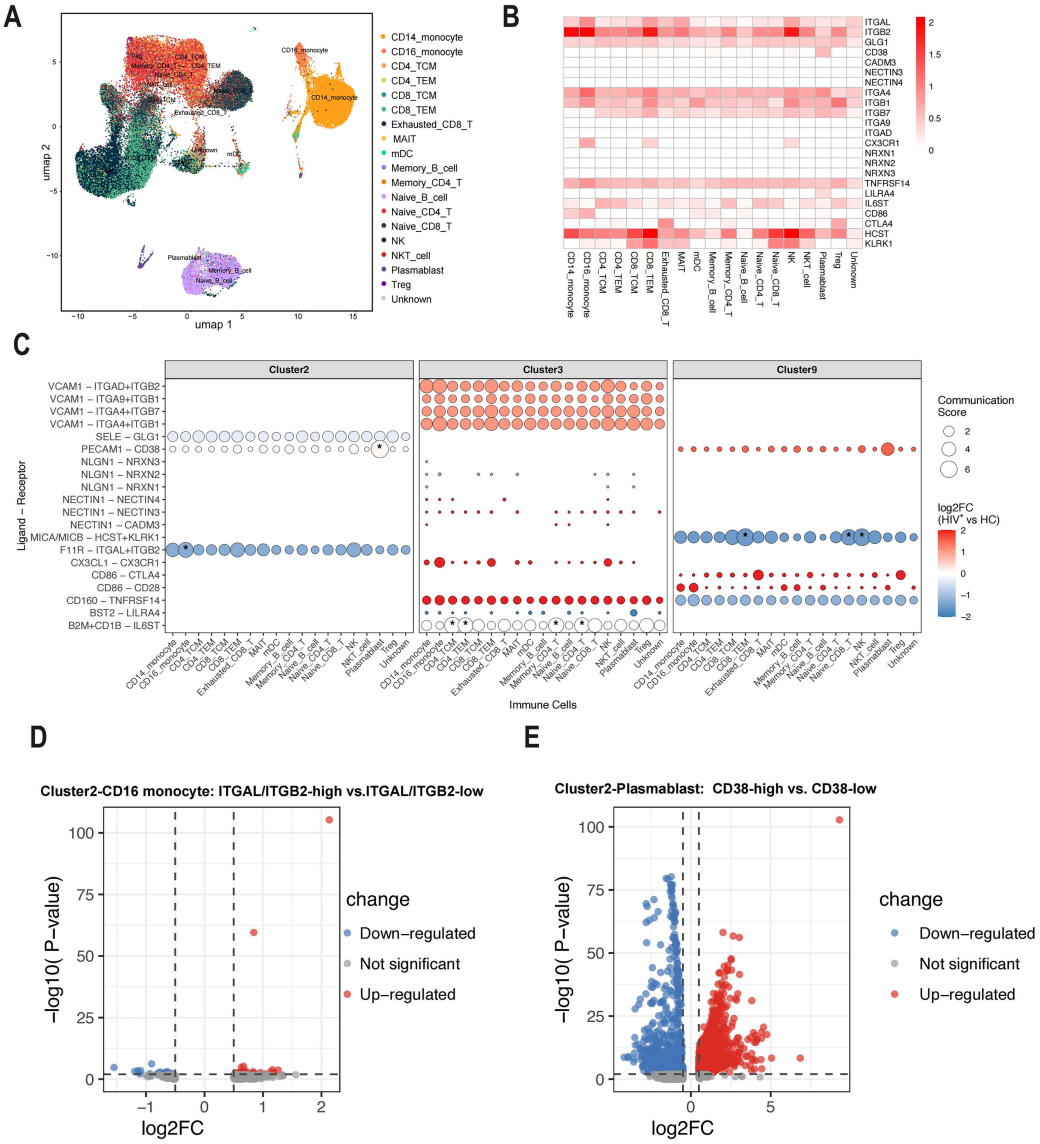
Receptor expression patterns and predicted EV-immune cell communication. **(A)** UMAP visualization of immune cell subpopulations from the GSE220790 dataset. **(B)** Heatmap showing the expression of receptors corresponding to EV-associated ligands from clusters 2, 3, and 9 across immune cell subsets. **(C)** Bubble plot illustrating predicted EV-immune cell interaction pairs identified through ligand-receptor analysis. *FDR <0.05. **(D-E)** Volcano plots showing differential gene expression between immune cell subsets stratified according to receptor expression levels potentially associated with Cluster 2 EVs interactions. **(D)** ITGAL^high^+ITGB2^high^ *vs.* ITGAL^low^+ITGB2^low^ CD16^+^ monocytes; **(E)** CD38^high^ *vs.* CD38^low^ plasmablasts. Up-regulated genes are shown in red, down-regulated genes in blue, and non-significant genes in gray.

To further examine the potential target immune cells and immunomodulatory mechanisms of the core HIV-associated EV subpopulations, CD31^+^ EVs (cluster 2), B2M^+^ EVs (cluster 3), and MUC16^+^ EVs (cluster 9), we analyzed immune-cell receptor expression and EV-mediated intercellular communication using a public single-cell RNA sequencing dataset. Clustering based on canonical marker-gene expression identified 18 immune cell subsets, including naive, memory, and effector CD4^+^ T cells; and naive, central memory, effector memory, and exhausted CD8^+^ T cell subsets; monocytes; dendritic cells; natural killer (NK) cells; and B cell subsets (**Figure 5A**). Cell-type annotation was supported by canonical lineage-specific marker gene expression (**Supplementary Figure 4A**). Receptors expression corresponding to key EV cluster ligands was then examined across immune cell subsets (**Figure 5B**). Integrin family receptors, including ITGAL and ITGB2, were predominantly expressed in monocytes and NK cells, whereas HCST showed relatively restricted expression in CD8^+^ TEM cells, providing molecular support for the predicted target-cell tropism of each EV subpopulation.

Based on ligand-receptor co-expression profiles, we calculated intercellular communication scores and constructed an interaction network between EV subpopulations and immune cells (**Figure 5C**). CD31^+^ EVs (cluster 2) showed predicted interactions with plasmablasts and CD16^+^ monocytes through EV-derived CD31 paired with cellular CD38 and EV-derived F11R paired with cellular ITGAL/ITGB2. B2M^+^ EVs (cluster 3) exhibited strong predicted with four CD4^+^ T cell subsets, including CD4^+^ TCM, CD4^+^ TEM, Naive CD4^+^ T, and Memory CD4^+^ T cells, through EV-derived B2M and CD1B with cellular IL6ST. MUC16^+^ EVs (cluster 9) preferentially interacted with CD8^+^ T cells and NK cells through EV-derived MICA/MICB and cellular HCST/KLRK1.

### Integrated pathway analysis suggests cell-type-specific immune reprogramming by EV subsets

**Figure 6:**
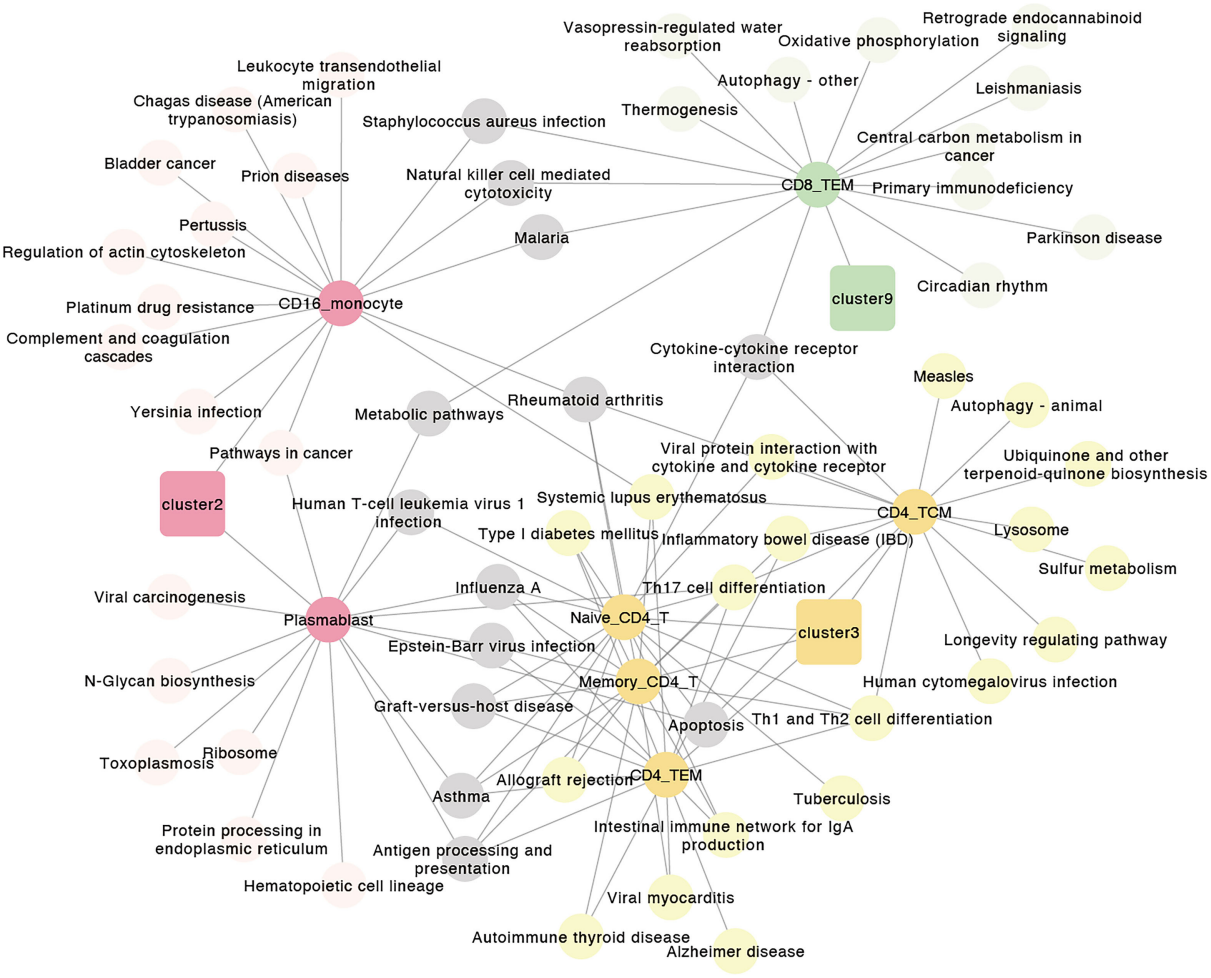
Integrated overview of KEGG pathways enriched in immune cell subsets predicted to interact with HIV-associated EV subclusters.

Cells were stratified into receptor-high and receptor-low groups according to the median module score. Differentially expressed genes (DEGs) analysis showed that receptor-high immune cells displayed distinct transcriptomic patterns (**Figures 5D, E**; **Supplementary Figures 4B, C**). CD31^+^ EVs (cluster 2) were predicted to target plasmablasts and CD16^+^ monocytes through CD38 and ITGAL/ITGB2, with 2011 DEGs identified in CD38^high^ vs. CD38^low^ plasmablasts and 62 in DEGs ITGAL^high^+ITGB2^high^ vs. ITGAL^low^+ITGB2^low^ CD16^+^ monocytes (**Figures 5D, E**). For B2M^+^ EVs (cluster 3), comparisons between IL6ST^high^ and IL6ST^low^ cells identified 54 DEGs in CD4 TCM, 85 DEGs in CD4 TEM, 70 DEGs in naive CD4^+^ T cells, and 118 DEGs in memory CD4+ T cells (**Supplementary Figure 4B**). For MUC16^+^ EVs (cluster 9), comparisons between HCST^high^+KLRK1^high^ and HCST^low^+KLRK1^low^ cells identified 37 DEGs in naive CD8^+^ T cells and 41 DEGs in NK cells (**Supplementary Figure 4C**). We constructed an integrated functional network summarizing KEGG pathway enrichment results across receptor-high immune cells targeted by EV clusters 2, 3, and 9 (**Figure 6**; **Supplementary Table 4**). CD31^+^ EVs (cluster 2) were mainly associated with pathways related to protein processing, metabolism, and viral infection in plasmablasts, as well as leukocyte transendothelial migration and immune regulation in CD16^+^ monocytes. B2M^+^ EVs (cluster 3) were associated with broad regulatory effects on CD4^+^ T cell subsets, involving T helper cell differentiation, antigen presentation, and autoimmune diseases, suggesting a potential role in shaping T cell immune responses during HIV infection. MUC16^+^ EVs (cluster 9) were linked to oxidative phosphorylation and natural killer cell-mediated cytotoxicity pathways in CD8^+^ TEM cells, suggesting a possible contribution to enhance antiviral effector functions. To illustrate these putative regulatory mechanisms, we further constructed a schematic working model depicting the divergent immunomodulatory roles of distinct EV subpopulations in HIV persistence. Overall, this network suggests that EVs may participate in divergent and cell-type-specific immunomodulatory programs, with CD31^+^ EVs-associated inflammatory reprogramming of CD16^+^ monocytes representing a candidate mechanism related to HIV-associated immune dysregulation.

## Discussion

HIV infection is characterized by latent viral reservoirs and persistent systemic immune dysregulation. In this exploratory study, we used PBA to characterize HIV-associated EV heterogeneity and identified functionally distinct EV subpopulations that may participate in cell-type-specific immune reprogramming, such as CD31^+^ EVs, B2M^+^ EVs, and MUC16^+^ EVs, which may participate in divergent immunomodulatory programs relevant to HIV persistence.Although AIDS is considered a proteopathy, the potential of EV protein content in the plasma of PLWH has only recently been unveiled. Prior studies suggest that HIV-infected cells may use EVs to modulate immune signaling by transferring Nef and Tat or host-derived immune regulators (11–13). This result extends previous observations with a conserved set of dysregulated proteins, including B2M, CD31, CD300E, EPS8, SELP, and TARM1, which were enriched in immune-related pathways, suggesting that EVs may act as systemic carriers of inflammatory and immune-regulatory signals. B2M and CD31 were increased in the HIV^+^ group in this study. Prior studies indicate that HIV infections increase the synthesis of β2-M (26), and increased β2-M levels are associated with inflammatory responses in tumors and viral infections (27). Together, these reports suggest that the prolonged state of hyperinflammation characterizes systemic chronic immune activation, which could result in elevated β2-M levels in PLWH (28, 29). Notably, increased β2-M correlates with poor survival in PLWH (29). Homotypic and heterotypic PECAM1 (CD31) pathways facilitate TGF-β–mediated suppression of T cell activity (30) and CD31 expression was associated with higher PD-1 and CD38/HLA-DR co-expression in the context of HIV-associated chronic immune activation (31). However, CD300E and EPS8 were decreased in HIV^+^ group. A study found a significant positive correlation between the expression of the activating receptor CD300E in monocytes with CD4^+^ T cell count, which decreased in patients whose viremia was controlled by undergoing ART (32).

LC-MS/MS proteomics (33, 34) has long been used to characterize exosome cargo in HIV-infected individuals. Instead of isolating EVs *via* size exclusion chromatography, the PBA detected membrane proteins (17). Here, we used the PBA technique to simultaneously profile approximately 543 types of membrane proteins at the single EV level in the plasma of HIV^+^ group and HC group. We observed three EV subpopulations, including CD31^+^ EVs, B2M^+^ EVs, and MUC16^+^ EVs, which showed distinct inferred cellular origins and ligand-receptor signatures and may contribute to divergent immunomodulatory programs relevant to HIV persistence. CD31^+^ EVs showed predicted interactions with CD16^+^ monocytes and plasmablasts through integrin-related ligand-receptor pairs, including EV-driven F11R paired with cellular ITGAL/ITGB2 and EV-driven CD31 paired with cellular CD38, and these interactions were associated with inflammatory signatures, leukocyte transendothelial migration, and metabolic reprogramming. As reported, CD31 is expressed on memory CD8^+^ T cells and associated with higher PD-1 and CD38/HLA-DR co-expression in the context of HIV-associated chronic immune activation (31). Meanwhile, CD38 overexpression could facilitate CD4 T cell depletion in HIV infection (35) and previous reports have shown that CD4^+^CD38^+^ TCM cells contribute to HIV persistence in HIV-infected individuals on long-term ART (36). In this study, the cellular origins of marker-positive EVs or receptors were integrated with public single-cell transcriptomes and analyzed using the CellMarker and CellPhoneDB databases. For example, CD31^+^ EVs are likely derived from endothelial cells, whereas CD38 expression predominantly marks plasmablasts. This finding is consistent with prior studies that endothelial cells contribute to HIV-associated inflammation (18, 37), and with previous reports showing that monocyte activation and plasmablast dysfunction are involved in HIV-related immune dysregulation (38–40). However, the present analysis identifies endothelial-derived EVs as candidate mediators rather than proven drivers of these processes. Ligand-receptor analysis suggested that B2M^+^ EVs engage CD4^+^ T cells through integrin-related ligands such as VCAM1 and SELE, and receptor-stratified transcriptomic analysis suggested that cluster 3-associated signaling may be linked to survival- and latency-related pathways in CD4^+^ TCM cells. Enrichment of T helper cell differentiation and antigen-presentation pathways further suggests that B2M^+^ EVs may modulate T-cell function in a manner relevant to viral persistence, potentially by supporting TCM cell survival or limiting effector differentiation. For MUC16^+^ EVs (cluster 9), predicted MICA/MICB-HCST/KLRK1 interactions were associated with cytotoxic function and oxidative phosphorylation in CD8^+^ TEM cells, which is consistent with previous reports that NK and CD8^+^ T-cell cytotoxicity contribute to viral control (41–43). During chronic HIV/SIV infection, abundantly produced G0 glycoform IgG binds MUC16 with high specificity and avidity, enriches IgG specific for viral envelope gp120 and gp41, and sequesters HIV virions in mucus to prevent invasion of mucosal cells (44). Similar regulatory effects of exosomes have also been verified in immune microenvironment modulation, which participates in vascular growth and bone tissue biological regulation (45). This potential functional complementarity among EV subpopulations suggests a complex EV-associated network in the HIV immune microenvironment.

Despite the interested findings in this study, several limitations of the study warrant acknowledgment. The sample size was small, the statistical power for differential proteomic analysis was limited, these findings should be interpreted as hypothesis-generating rather than definitive. Furthermore, we could not directly resolve the exact cellular sources of CD31^+^ EVs, B2M^+^ EVs, and MUC16^+^ EVs, nor did we perform direct validation experiments to assess the function of these EV subpopulations. Such limitations may compromise the generalizability and robustness of the research findings. Additional limitations include the lack of longitudinal data to track dynamic changes in EV subpopulations during HIV disease progression or ART treatment, and the absence of mechanistic studies to confirm the causal relationship between EV subpopulation interactions and immune dysregulation/reservoir persistence. Notably, our study is the first to use PBA technology to profile EV membrane proteins at the single-EV level in PLWH, which overcomes the limitation of traditional bulk proteomics that masks EV heterogeneity.

In summary, our findings propose an EV-associated immunoregulatory network in HIV infection. HIV infection appears to remodel circulating EV subpopulations rather than simply altering bulk EV quantity. CD31^+^ EVs (cluster 2) may contribute to systemic immune dysregulation by interacting with CD16^+^ monocytes and plasmablasts, whereas B2M^+^ EVs may be associated with CD4^+^ TCM survival, and MUC16^+^ EVs may be linked to antiviral cytotoxicity. This pattern connects endothelial dysfunction, inflammatory activation, and reservoir-associated immune changes, but it should be interpreted cautiously because the present study is exploratory and based on a limited sample size. Future studies using larger cohorts, longitudinal sampling, EV isolation from defined cellular sources, and functional perturbation experiments are required to confirm the biological relevance of these candidate mechanisms.

## Conclusion

This study suggests that HIV infection selectively reshapes EVs subpopulation composition without substantially altering bulk EV abundance. CD31^+^ EVs (cluster 2) may represent a candidate EV subset associated with immune dysregulation, with predicted communication involving CD16^+^ monocytes and plasmablasts through ITGAL/ITGB2 and CD38, and enrichment of inflammatory and migration-related pathways. Together with HIV-enriched EV subpopulations linked to CD4^+^ TCM reservoir cells (cluster 3) and CD8^+^ TEM/NK cells (cluster 9), these findings support EV heterogeneity as a potentially important feature of HIV-associated immune dysfunction and latent reservoir persistence. Functional validation and larger clinical cohorts are needed to confirm these proposed mechanisms.

## Data Availability

The original contributions presented in the study are included in the article/**Supplementary Material**. Further inquiries can be directed to the corresponding authors.
