# Supplementary material for: Proteomic profiling of single extracellular vesicles reveals association of CD31+ EV subpopulation with immune dysregulation in people living with HIV
Source: Front Immunol. 2026 Jul 7;17:1871641. doi: 10.3389/fimmu.2026.1871641 (PMC13384927; doi:10.3389/fimmu.2026.1871641)
Supplement: Supplementary file 1 [file DataSheet1.pdf]

Supplementary Figure S1. Schematic workflow of this exploratory study.

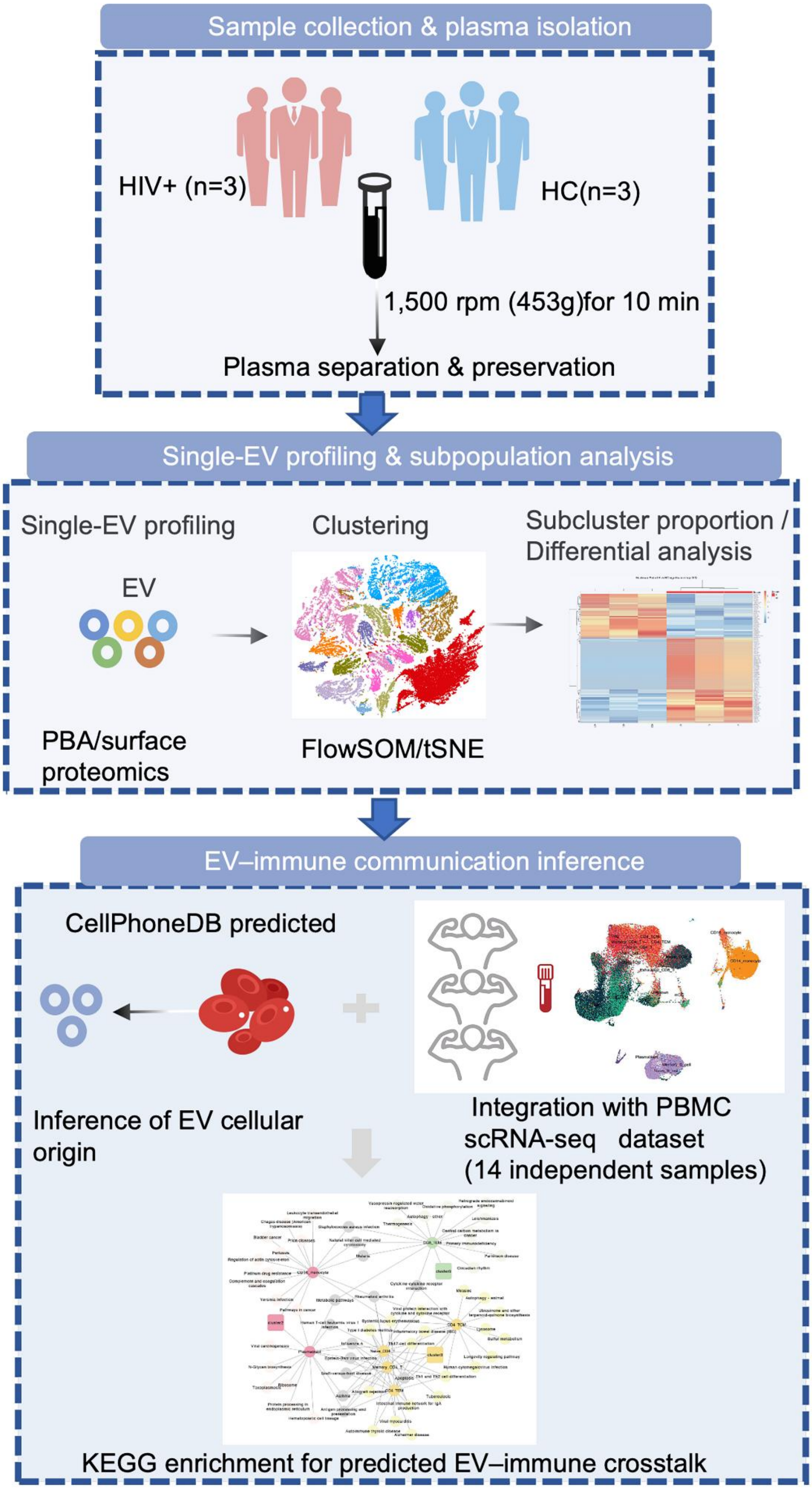

Supplementary Figure S2. GO functional enrichment analysis of EV membrane proteins.

(A) Top 10 significantly enriched terms for Biological Process (BP). $p < 0.05$.

(B) Top 10 significantly enriched terms for Cellular Component (CC). $p < 0.05$.

(C) Top 10 significantly enriched terms for Molecular Function (MF). $p < 0.05$.

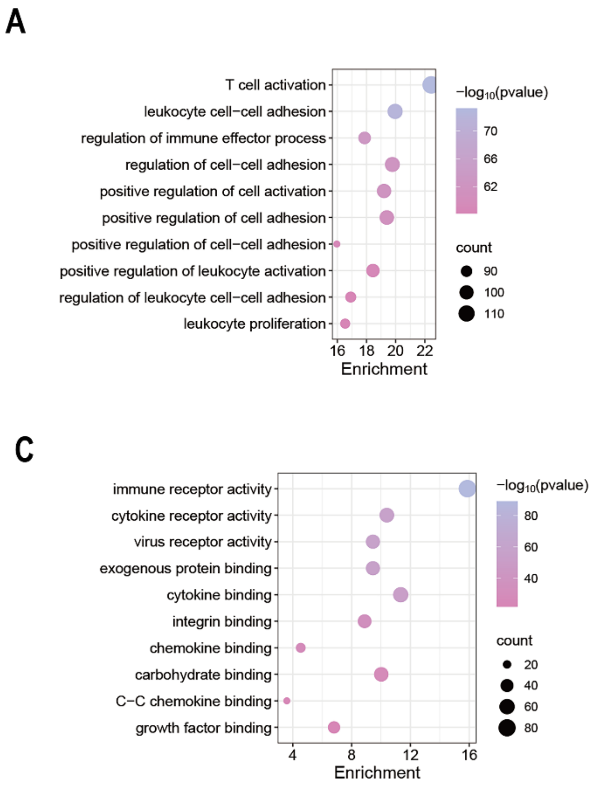

Supplementary Figure S3. Characterization of EV cluster distributions.

(A) Bar plots showing the relative abundance of 12 EV clusters in HIV^+ group and HC group.

(B) UMAP visualization illustrating the distribution of the 12 clusters across 6 individual samples. Each color represents a distinct cluster.

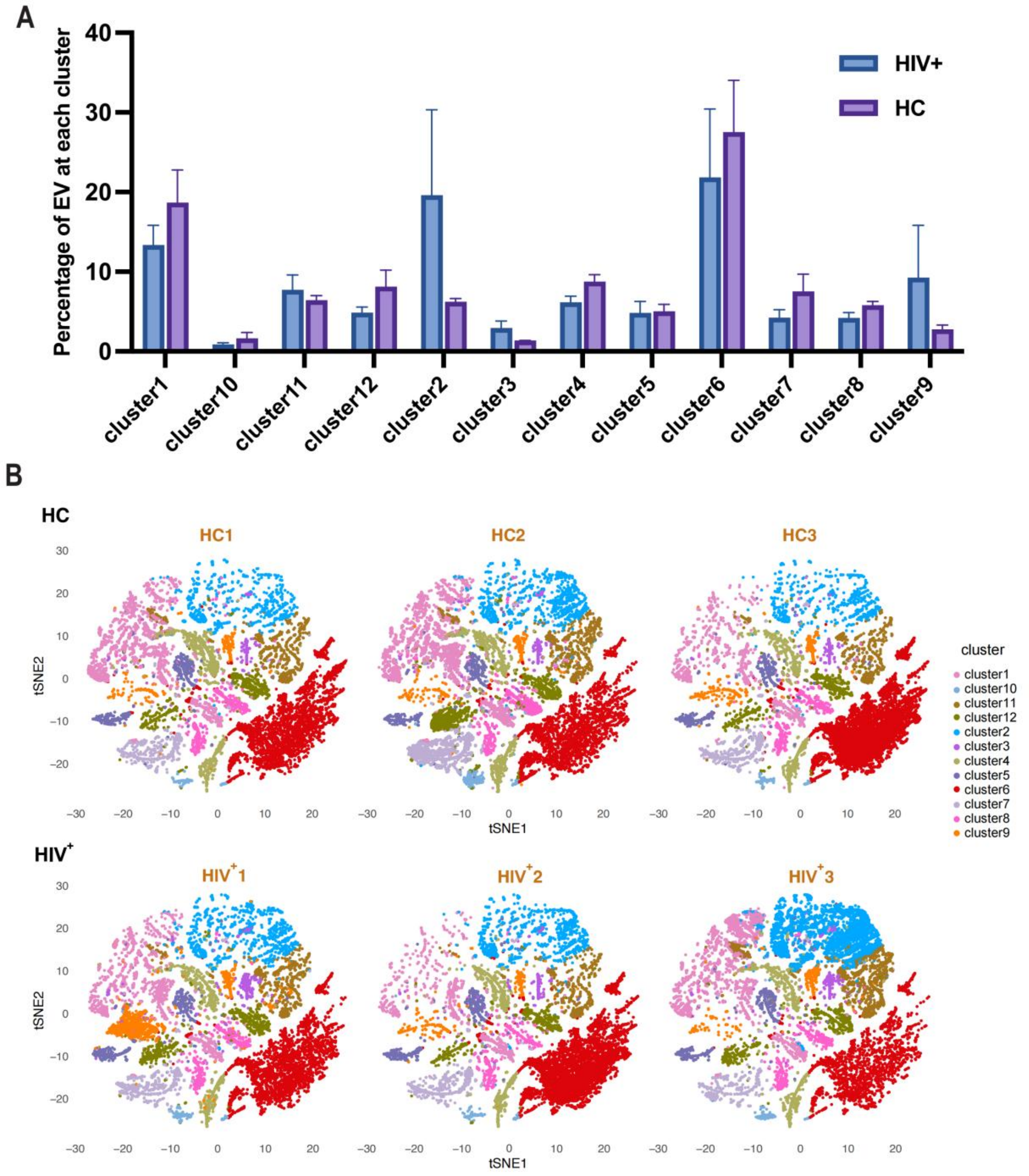

Supplementary Figure S4. Immune cell marker validation and receptor expression heatmaps for EV subpopulations.

(A) Bubble plot of canonical marker genes used for cell-type annotation of single-cell immune populations from the GSE220790 dataset; (B) Volcano plots showing differential gene expression analyses in four CD4+ T-cell subsets stratified by IL6ST expression level (IL6ST^{high} vs. IL6ST^{low}), based on receptors corresponding to EV-associated ligands from Cluster 3; (C) Volcano plots showing differential gene expression analyses in NK cells and CD8+ effector memory T cells stratified by HCST/KLRK1 expression level (HCST^{high}+KLRK1^{high} vs. HCST^{low}+KLRK1^{low}), based on receptors corresponding to EV-associated ligands from Cluster 9.

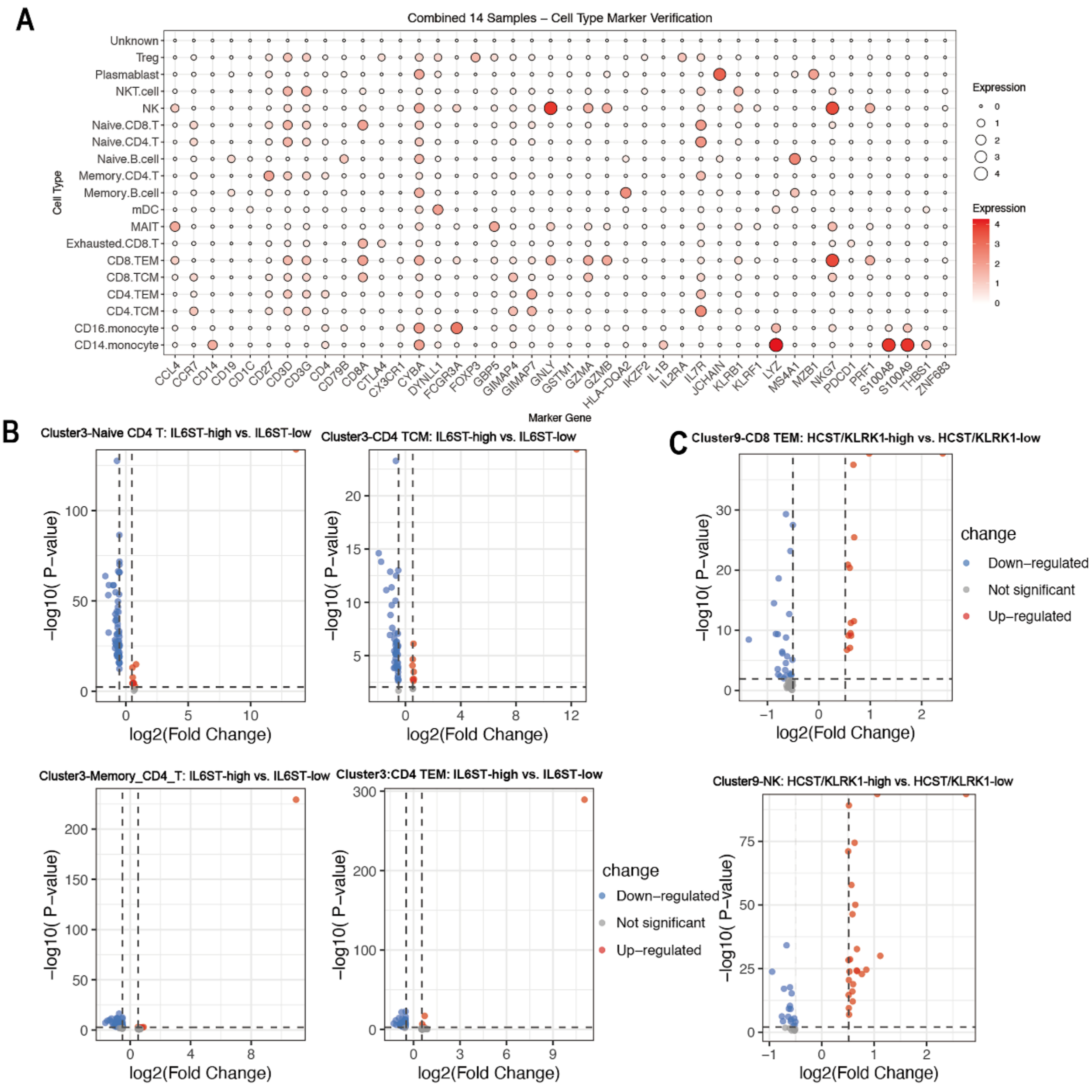

Supplementary Table S1. Summary of sequencing quality.

Sample ID	Clean Q20(%)	Total Reads	Mapped Reads	Unique Reads	EV Count	Mapped Reads/Unique Reads	Protein/EV Count
H1	100.00%	15107213	13280217	3089610	688688	4.298	4.486
H2	100.00%	15997404	14075575	3592027	724879	3.919	4.955
H3	100.00%	17719446	15637492	3589368	677842	4.357	5.295
HC1	100.00%	16593597	14553571	3259362	722693	4.465	4.51
HC2	100.00%	16024885	14100502	3462745	718505	4.072	4.819
HC3	100.00%	17857707	15719702	4045043	834534	3.886	4.847

Abbreviations: EV, extracellular vesicle; QC, quality control.

Supplementary Table S2. Featured proteins in EV clusters.

cluster	EV marker	Frequency
cluster1	ADIPOQ	0.7
cluster2	PECAM1	1
cluster3	B2M	1
cluster4	BIRC5	0.62
cluster5	CD8A	0.53
	CEACAM8	0.51
cluster6	-	-
cluster7	CD63	1
cluster8	CD36	0.54
cluster9	MUC16	0.72
cluster10	CD3E	0.99
cluster11	ITGB3	1
cluster12	CDH6	0.53
	CTLA4	0.51

Supplementary Table S3. Ligand-receptor interaction analysis for clusters 2, 3, and 9.

cluster	EV_Protein	ligand	receptor
cluster2	SELE	CEACAM1	SELE
cluster2	F11R	F11R	ITGAL+ITGB2
cluster2	CCR9	CCL25	CCR9
cluster2	SELE	PODXL2	SELE
cluster2	SELP	PODXL2	SELP
cluster2	GLP1R	GCG	GLP1R
cluster2	SELE	SELE	GLG1
cluster2	SELE	SELPLG	SELE
cluster2	SELP	SELPLG	SELP
cluster2	SSTR2	CORT	SSTR2
cluster2	SSTR2	SST	SSTR2
cluster2	SELP	CD24	SELP
cluster2	SELP	CD34	SELP

cluster2	SELE	CD44	SELE
cluster2	PECAM1	PECAM1	CD38
cluster2	PECAM1	CD177	PECAM1
cluster3	NECTIN1	NECTIN1	CADM3
cluster3	NECTIN1	NECTIN1	NECTIN3
cluster3	NECTIN1	NECTIN1	NECTIN4
cluster3	VCAM1	VCAM1	ITGA4+ITGB1
cluster3	VCAM1	VCAM1	ITGA4+ITGB7
cluster3	VCAM1	VCAM1	ITGA9+ITGB1
cluster3	VCAM1	VCAM1	ITGAD+ITGB2
cluster3	CX3CL1	CX3CL1	CX3CR1
cluster3	NLGN1	NLGN1	NRXN1
cluster3	NLGN1	NLGN1	NRXN2
cluster3	NLGN1	NLGN1	NRXN3
cluster3	SELP	PODXL2	SELP
cluster3	SELP	SELPLG	SELP
cluster3	CD160	CD160	TNFRSF14
cluster 3	TNFRSF25	TNFSF12	TNFRSF25
cluster3	TNFRSF25	TNFSF15	TNFRSF25
cluster3	BST2	BST2	LILRA4
cluster3	SELP	CD24	SELP
cluster3	SELP	CD34	SELP
cluster3	CD2	CD58	CD2
cluster3	NECTIN1	CD96	NECTIN1
cluster9	IGF1R	IGF1	IGF1R
cluster9	IGF1R	IGF2	IGF1R
cluster9	NOTCH2	CNTN1	NOTCH2
cluster9	NOTCH2	DLK1	NOTCH2
cluster9	NOTCH2	DLL1	NOTCH2
cluster9	NOTCH2	DLL3	NOTCH2
cluster9	NOTCH2	DLL4	NOTCH2
cluster9	NOTCH2	JAG1	NOTCH2
cluster9	NOTCH2	JAG2	NOTCH2
cluster9	NRP1	PGF	NRP1
cluster9	NRP1	SEMA3A	NRP1
cluster9	CD160	CD160	TNFRSF14
cluster 9	TNFRSF13C	TNFSF13B	TNFRSF13C
cluster9	NRP1	VEGFA	NRP1
cluster9	NRP1	VEGFB	NRP1
cluster9	CD28	CD80	CD28
cluster9	CD86	CD86	CD28
cluster9	CD86	CD86	CTLA4
cluster 9	MICA	MICA	HCST+KLRK1
cluster9	MICB	MICB	HCST+KLRK1

cluster9	PECAM1	PECAM1	CD38
cluster9	PECAM1	CD177	PECAM1

Supplementary Table S4. KEGG pathways enriched in immune cell subsets associated EV subclusters.

Cluster	Cell type	KEGG pathways	ID	Input number	P-value	Input gene
cluster2	Plasmablast	Ribosome	hsa03010	90	3.17E-55	RPL23A|RPL22|MRPS2|RPL14|RPL15|RPL17|RPL10|RPL11|RPL12|RPL13|RPL18|RPL19|UBA52|UBC|MRPL27|RPL37A|RPL8|RPL9|RPL6|RPL7|RPL4|RPL5|RPL3|RPL7A|RPS9|RPL10A|MRPL12|MRPL15|MRPL17|RSL24D1|RPS12|RPLP2|RPLP1|RPL18A|RPL35A|RPS15A|RPS7|RPS6|RPS5|RPS3|RPL41|RPS8|RPS4Y1|RPS27L|RPL13A|RPL27A|RPS26|RPS27|RPS24|RPS27A|RPS23|RPS20|RPS21|MRPS18A|RPS28|RPS29|RPS4X|RPSA|RPS3A|RPS13|RPL36A|RPS11|RPS10|RPS16|RPS15|RPS14|RPS19|RPS18|MRPS16|MRPS14|MRPS11|RPL29|RPL28|RPL24|RPL27|RPL26|RPL21|RPL23|FAU|RPS25|RPL26L1|RPL38|RPL39|RPL36|RPL37|RPL34|RPL35|RPL32|RPL30|RPL31
cluster2	Plasmablast	Protein processing in endoplasmic reticulum	hsa04141	72	9.70E-38	RPN1|GANAB|UBE2J1|HSPA5|NPLOC4|HSPA8|CALR|DDOST|LMAN2|STT3B|DERL1|EIF2S1|ERO1A|DAD1|SAR1B|CANX|XBP1|SYVN1|HERPUD1|SEC23B|SKP1|SELENOS|TXNDC5|DERL2|MAP3K5|HSP90AB1|DNAJB12|DNAJB11|PREB|CAPN2|UBE2G1|UBXN6|LMAN1|ERLEC1|UGGT1|TRAM1|SEL1L|UBE4B|PDIA6|P4HB|PDIA4|PDIA3|MAN1A1|DNAJC3|SEC24D|EDEM2|EDEM3|EDEM1|SEC13|SEC61G|STT3A|RRBP1|MAN1A2|SIL1|MOGS|MBTPS1|DERL3|EIF2AK3|HYOU1|DNAJC1|HSP90B1|BAK1|PPP1R15A|SSR1|SSR2|SSR3|SSR4|SEC24A|ATF4|WFS1|SEC61A1|CKAP4
cluster2	Plasmablast	Metabolic pathways	hsa01100	174	4.26E-24	PCK2|RPN1|MT-CO2|MT-CO3|AGK|MT-CO1|NDUFB7|IDI1|NDUFB3|POMT1|ACADVL|ALG8|PMM2|PYCR1|UQCRB|NDUFAB1|DCK|MT-ND6|PIK3CD|SELENOI|NANS|MPST|ALG9|MAN1A2|PLD4|ENTPD1|IDH2|ALOX5|IMPA1|PHGDH|MCEE|SHMT1|NME4|INPP5D|PLD3|OAT|PIP5K1B|CHAC2|C1GALT1C1|UGCG|VDR|ACADS|ALG3|ALG1|ALG6|GAPDH|ALG

						5|MGST2|PPOX|AKR1A1|IMPAD1|PTGDS|TST|CYP2U1|GLDC|GMDS|MRI1|B4GALT1|B4GALT3|HSD17B10|HMBS|PIKFYVE|MT-CYB|ADA2|UAP1|NUDT5|MAN1A1|PGM3|GYS1|TK2|TK1|STT3B|STT3A|ACO2|ETHE1|MT-ND5|HACD3|MTHFD2|ACAD8|ATP5F1E|MOGS|G6PD|GAB1|ATP5F1A|HMOX2|ELOVL5|DTYMK|ATP6V0A1|ATP6V1G1|CRLS1|GFPT1|MINPP1|GCSH|GANAB|AGPS|EARS2|PDHA1|IDS|PDXK|SMPD1|SMPD3|GLS|NDUFV3|CD38|AFMID|PYGB|TECR|ALAS1|ALDH18A1|DPM3|DPM2|PGM1|ASL|PPCDC|DPAGT1|UGDH|ACSS1|GMPPA|GMPPB|COX4I1|MVD|ALG12|SLC33A1|ALG14|ADH5|GSTM1|GPT2|PGLS|ASNS|ALDH2|PPAT|GGCX|OXCT2|PDE4B|HIBCH|ATP6V0C|CBLB|DHFR|HYI|CAT|DDOST|RRM2|ATP5MC2|DAD1|CAD|PAPSS1|CBR1|HSD17B8|LPCAT4|LPCAT1|EPRS|LDHA|GPX4|PIGB|CANT1|ST3GAL4|NUDT2|PIGK|PIGQ|PIGS|SRM|UPP1|INPP1|APRT|PFKFB2|PMVK|TXNDC12|PSAT1|TYMS|EBP|NDUFS6|COQ5|CHPF|FUT8
cluster2	Plasmablast	Pathways in cancer	hsa05200	85	1.71E-18	BCL2L1|RASGRP3|RASGRP1|CSF2RA|TGFBR2|CDKN1B|LAP3|NFKBIA|BIRC3|HIF1A|IL6ST|IFNAR2|MAP2K2|FOXO1|CDKN2A|IFNGR1|PMAIP1|IGF1|PIK3CD|FLT3|LAMA5|STAT6|RHOA|NCOA1|STAT3|CALM3|NCOA4|CALM1|IL12RB1|GADD45B|TPM3|CKS1B|EML4|RPS6KB2|HSP90AB1|DVL3|JUP|CALM2|CXCR4|HDAC2|MGST2|TXNRD2|BIRC5|KEAP1|ITGB1|TCF4|TRAF3|TRAF4|TRAF5|FOS|GNB5|CKS2|PRKCB|JAK1|RXRB|IL6R|IL4R|COL4A4|IL3RA|HSP90B1|VEGFB|SLC2A1|ZBTB17|MDM2|SMAD3|EGLN3|E2F2|SKP1|CASP7|EGLN1|CASP3|SOS1|ESR1|GSTM1|CCND3|CCND2|NCOA3|NOTCH2|BAK1|F2R|RUNX1|CDK6|PTCH2|IL15RA|CDC42
cluster2	Plasmablast	Epstein-Barr virus infection	hsa05169	48	1.27E-16	CALR|RPN2|HDAC2|CDKN1B|NFKBIA|PSMD1|HLA-F|USP7|IFNAR2|TRAF5|HLA-DRA|STAT3|GADD45B|PIK3CD|OAS1|ISG15|ICAM1|JAK1|RE

						LB|LYN|IRAK1|ADRM1|PSMC6|HLA-DPB1|TRAF3|CDK6|ENTPD1|CD44|HLA-DRB1|PDIA3|HLA-DRB5|MDM2|E2F2|IRF3|CASP3|SYK|CCND3|CCND2|BAK1|ITGAL|HLA-DOA|HLA-DOB|HLA-DPA1|CD19|HLA-DMB|HLA-DQA1|HLA-DQA2|HLA-DMA
cluster2	Plasmablast	Human T-cell leukemia virus 1 infection	hsa05166	48	2.21E-15	BCL2L1|CALR|MAD2L1|TGFBR2|NFKBIA|HLA-F|SMAD3|CD4|MAP2K2|VDAC1|CANX|DLG1|HLA-DRA|MAML2|PIK3CD|TNFRSF13C|ICAM1|JAK1|RELB|HLA-DPB1|CDKN2A|FOS|HLA-DRB1|CREB3|SLC25A6|SLC25A4|PTTG1|HLA-DRB5|ANAPC5|NRP1|CCNB2|E2F2|IL15RA|CCND3|CCND2|SLC2A1|CDC20|CREB3L2|ZFP36|ITGAL|HLA-DOA|HLA-DOB|HLA-DPA1|ATF4|HLA-DMB|HLA-DQA1|HLA-DQA2|HLA-DMA
cluster2	Plasmablast	Viral carcinogenesis	hsa05203	43	1.28E-13	HDAC2|PMAIP1|HDAC7|CDKN1B|NFKBIA|HLA-F|IL6ST|CDKN2A|GTF2A1|REL|CDC20|MAPKAPK2|GSN|DLG1|HIST4H4|STAT3|PIK3CD|HNRNPK|JAK1|LYN|YWHAG|YWHAE|YWHAZ|TRAF3|TRAF5|RHOA|CREB3|MRPS18B|MDM2|USP7|HIST1H2BH|HIST1H2BJ|IRF3|CASP3|SYK|CCND3|CCND2|BAK1|CREB3L2|CDK6|ATF4|SP100|CDC42
cluster2	Plasmablast	N-Glycan biosynthesis	hsa00510	23	2.10E-13	RPN1|GANAB|ALG9|ALG3|ALG1|ALG6|ALG8|ALG5|DDOST|DAD1|STT3A|B4GALT1|B4GALT3|DPM3|DPM2|DPAGT1|STT3B|ALG12|ALG14|MAN1A1|MAN1A2|MOGS|FUT8
cluster2	Plasmablast	Th17 cell differentiation	hsa04659	31	4.31E-13	TGFBR2|IL6ST|NFKBIA|HIF1A|SMAD3|CD4|IFNGR1|STAT6|HLA-DRA|STAT3|IL12RB1|RORA|HSP90AB1|ZAP70|JAK1|HLA-DPB1|IL4R|FOS|HLA-DRB1|RXRB|IL6R|TIFA|HLA-DRB5|RUNX1|HLA-DOA|HLA-DOB|HLA-DPA1|HLA-DMB|HLA-DQA1|HLA-DQA2|HLA-DMA
cluster2	Plasmablast	Apoptosis	hsa04210	34	1.13E-12	BCL2L1|TNFSF10|PMAIP1|BIRC5|NFKBIA|BIRC3|CASP10|MAP2K2|EIF2S1|HTRA2|DAXX|ITPR1|TUBA1A|PIK3CD|MAP3K5|CFLAR|CAPN2|FOS|CTSB|PARP1|PARP2|IL3RA|CTSS|CASP6|CASP7|LMNB1|CASP3|GZMB|BCL2A1|EIF2AK3|BAK1|ATF4|AIFM1|GADD45B

cluster2	Plasmablast	Hematopoietic cell lineage	hsa04640	28	6.20E-12	CD38|CSF2RA|CD55|CD59|ITGA4|CD36|CD37|CD4|FLT3|HLA-DRA|TFRC|CD22|HLA-DPB1|IL4R|CD44|HLA-DRB1|IL6R|IL3RA|HLA-DRB5|HLA-DOA|HLA-DOB|HLA-DPA1|CD24|CD19|HLA-DMB|HLA-DQA1|HLA-DQA2|HLA-DMA
cluster2	Plasmablast	Influenza A	hsa05164	36	1.01E-11	TNFSF10|NFKBIA|IFNAR2|MAP2K2|IFNGR1|VDAC1|CPSF4|EIF2S1|HLA-DRA|CIITA|PIK3CD|PRKCB|OAS1|ICAM1|JAK1|HLA-DPB1|TRAF3|HLA-DOB|HLA-DRB1|SLC25A6|HLA-DRB5|SLC25A4|IRF3|CASP3|SOCS3|CASP1|CCND3|DNAJC3|BAK1|HLA-DOA|CDK6|HLA-DPA1|HLA-DMB|HLA-DQA1|HLA-DQA2|HLA-DMA
cluster2	Plasmablast	Antigen processing and presentation	hsa04612	24	5.24E-11	CD74|HSPA5|HSPA8|HLA-F|CD4|CANX|HLA-DRA|CIITA|HSP90AB1|HLA-DPB1|PDIA3|CTSB|HLA-DRB1|PSME2|HLA-DRB5|CTSS|CALR|HLA-DOA|HLA-DOB|HLA-DPA1|HLA-DMB|HLA-DQA1|HLA-DQA2|HLA-DMA
cluster2	Plasmablast	Asthma	hsa05310	16	2.56E-10	HLA-DPB1|FCER1G|FCER1A|HLA-DMB|RAF1|HLA-DRB1|HLA-DRA|HLA-DRB5|HLA-DOA|HLA-DOB|HLA-DPA1|MS4A1|MBP|HLA-DQA1|HLA-DQA2|HLA-DMA
cluster2	Plasmablast	Toxoplasmosis	hsa05145	27	5.43E-10	BCL2L1|IL10RA|HSPA8|BIRC3|IFNGR1|HLA-DRA|STAT3|CIITA|JAK1|IRAK1|ITGB1|HLA-DPB1|NFKBIA|LAMA5|ALOX5|HLA-DRB1|HLA-DRB5|LDLR|SOCS1|CASP3|HLA-DOA|HLA-DOB|HLA-DPA1|HLA-DMB|HLA-DQA1|HLA-DQA2|HLA-DMA
cluster2	CD16_monocyte	Natural killer cell mediated cytotoxicity	hsa04650	5	2.16E-06	SH2D1B|VAV1|ITGB2|ITGAL|GZMB
cluster2	CD16_monocyte	Staphylococcus aureus infection	hsa05150	4	4.67E-06	ITGB2|ITGAL|C1QB|C1QA
cluster2	CD16_monocyte	Regulation of actin cytoskeleton	hsa04810	5	2.18E-05	VAV1|ARHGEF7|ACTN1|ITGB2|ITGAL
cluster2	CD16_monocyte	Prion diseases	hsa05020	3	2.75E-05	C1QB|C1QA|CCL5
cluster2	CD16_monocyte	Leukocyte transendothelial migration	hsa04670	4	3.09E-05	VAV1|ACTN1|ITGB2|ITGAL

cluster2	CD16_monocyte	Malaria	hsa05144	3	7.09E-05	ITGB2|ITGAL|CD36
cluster2	CD16_monocyte	Pathways in cancer	hsa05200	6	1.68E-04	RASSF1|CDKN1A|MGST2|GSTA4|CSF3R|FH
cluster2	CD16_monocyte	Platinum drug resistance	hsa01524	3	2.20E-04	GSTA4|MGST2|CDKN1A
cluster2	CD16_monocyte	Pertussis	hsa05133	3	2.46E-04	ITGB2|C1QB|C1QA
cluster2	CD16_monocyte	Complement and coagulation cascades	hsa04610	3	2.75E-04	ITGB2|C1QB|C1QA
cluster2	CD16_monocyte	Rheumatoid arthritis	hsa05323	3	4.11E-04	ITGB2|ITGAL|CCL5
cluster2	CD16_monocyte	Chagas disease (American trypanosomiasis)	hsa05142	3	5.83E-04	C1QB|C1QA|CCL5
cluster2	CD16_monocyte	Yersinia infection	hsa05135	3	9.20E-04	VAV1|ARHGEF7|TBK1
cluster2	CD16_monocyte	Systemic lupus erythematosus	hsa05322	3	1.20E-03	ACTN1|C1QB|C1QA
cluster2	CD16_monocyte	Bladder cancer	hsa05219	2	1.99E-03	RASSF1|CDKN1A
cluster3	Memory_CD4_T	Influenza A	hsa05164	10	1.68E-10	CASP1|HLA-DPB1|HLA-DRA|CCL5|FAS|HLA-DRB1|HLA-DRB5|HLA-DPA1|PYCARD|EIF2S1
cluster3	Memory_CD4_T	Allograft rejection	hsa05330	6	4.11E-09	HLA-DPB1|HLA-DRA|FAS|HLA-DRB1|HLA-DRB5|HLA-DPA1
cluster3	Memory_CD4_T	Graft-versus-host disease	hsa05332	6	6.20E-09	HLA-DPB1|HLA-DRA|FAS|HLA-DRB1|HLA-DRB5|HLA-DPA1
cluster3	Memory_CD4_T	Antigen processing and presentation	hsa04612	7	6.80E-09	CD74|HLA-DPB1|HLA-DRA|CTSB|HLA-DRB1|HLA-DRB5|HLA-DPA1
cluster3	Memory_CD4_T	Type I diabetes mellitus	hsa04940	6	8.03E-09	HLA-DPB1|HLA-DRA|FAS|HLA-DRB1|HLA-DRB5|HLA-DPA1
cluster3	Memory_CD4_T	Epstein-Barr virus infection	hsa05169	9	1.60E-08	HLA-DPB1|HLA-DRA|FAS|GADD45B|HLA-DRB1|HLA-DRB5|MAP2K3|ISG15|HLA-DPA1
cluster3	Memory_CD4_T	Autoimmune thyroid disease	hsa05320	6	2.53E-08	HLA-DPB1|HLA-DRA|FAS|HLA-DRB1|HLA-DRB5|HLA-DPA1
cluster3	Memory_CD4_T	Th17 cell differentiation	hsa04659	7	5.74E-08	HLA-DPB1|HLA-DRA|HLA-DRB1|HLA-DRB5|HLA-DPA1|IL6ST|GATA3
cluster3	Memory_CD4_T	Asthma	hsa05310	5	7.78E-08	HLA-DPA1|HLA-DPB1|HLA-DRA|HLA-DRB1|HLA-DRB5
cluster3	Memory_CD4_T	Inflammatory bowel disease (IBD)	hsa05321	6	7.80E-08	HLA-DPB1|HLA-DRA|HLA-DRB1|HLA-DRB5|HLA-DPA1|GATA3
cluster3	Memory_CD4_T	Systemic lupus erythematosus	hsa05322	7	2.35E-07	HLA-DPB1|HLA-DRA|ACTN4|HLA-DRB1|HLA-DRB5|HLA-DPA1|SNRPB

cluster3	Memory_CD4_T	Rheumatoid arthritis	hsa05323	6	5.05E-07	HLA-DPB1|HLA-DRA|CCL5|HLA-DRB1|HLA-DRB5|HLA-DPA1
cluster3	Memory_CD4_T	Th1 and Th2 cell differentiation	hsa04658	6	5.37E-07	HLA-DPB1|HLA-DRA|HLA-DRB1|HLA-DRB5|HLA-DPA1|GATA3
cluster3	Memory_CD4_T	Intestinal immune network for IgA production	hsa04672	5	6.25E-07	HLA-DPA1|HLA-DPB1|HLA-DRA|HLA-DRB1|HLA-DRB5
cluster3	Memory_CD4_T	Viral myocarditis	hsa05416	5	1.59E-06	HLA-DPA1|HLA-DPB1|HLA-DRA|HLA-DRB1|HLA-DRB5
cluster3	Naive_CD4_T	Th17 cell differentiation	hsa04659	6	5.57E-08	HLA-DPB1|IL2RB|TGFBR2|HLA-DRB1|IL6ST|HLA-DPA1
cluster3	Naive_CD4_T	Human T-cell leukemia virus 1 infection	hsa05166	6	3.17E-06	HLA-DPB1|IL2RB|TGFBR2|HLA-DRB1|ZFP36|HLA-DPA1
cluster3	Naive_CD4_T	Antigen processing and presentation	hsa04612	4	1.38E-05	CD74|HLA-DPB1|HLA-DRB1|HLA-DPA1
cluster3	Naive_CD4_T	Influenza A	hsa05164	5	1.45E-05	HLA-DPA1|HLA-DPB1|HLA-DRB1|TRADD|CCL5
cluster3	Naive_CD4_T	Cytokine-cytokine receptor interaction	hsa04060	6	1.63E-05	TGFBR2|IL2RB|CCL5|IL6ST|CCR6|TNFRSF4
cluster3	Naive_CD4_T	Tuberculosis	hsa05152	5	2.01E-05	CD74|HLA-DPB1|HLA-DPA1|HLA-DRB1|TRADD
cluster3	Naive_CD4_T	Rheumatoid arthritis	hsa05323	4	2.60E-05	HLA-DPA1|HLA-DPB1|HLA-DRB1|CCL5
cluster3	Naive_CD4_T	Th1 and Th2 cell differentiation	hsa04658	4	2.71E-05	HLA-DPA1|HLA-DPB1|IL2RB|HLA-DRB1
cluster3	Naive_CD4_T	Asthma	hsa05310	3	3.11E-05	HLA-DPA1|HLA-DPB1|HLA-DRB1
cluster3	Naive_CD4_T	Viral protein interaction with cytokine and cytokine receptor	hsa04061	4	3.70E-05	IL2RB|CCL5|CCR6|IL6ST
cluster3	Naive_CD4_T	Allograft rejection	hsa05330	3	5.49E-05	HLA-DPA1|HLA-DPB1|HLA-DRB1
cluster3	Naive_CD4_T	Graft-versus-host disease	hsa05332	3	6.79E-05	HLA-DPA1|HLA-DPB1|HLA-DRB1
cluster3	Naive_CD4_T	Type I diabetes mellitus	hsa04940	3	7.77E-05	HLA-DPA1|HLA-DPB1|HLA-DRB1
cluster3	Naive_CD4_T	Intestinal immune network for IgA production	hsa04672	3	1.12E-04	HLA-DPA1|HLA-DPB1|HLA-DRB1
cluster3	Naive_CD4_T	Apoptosis	hsa04210	4	1.18E-04	LMNA|PMAIP1|TRADD|ERN1
cluster3	CD4_TCM	Viral protein interaction with cytokine and cytokine receptor	hsa04061	4	1.23E-05	IL2RB|CCL5|CCR6|IL6ST

cluster3	CD4_TCM	Cytokine-cytokine receptor interaction	hsa04060	5	5.25E-05	TNFRSF4|IL2RB|CCL5|CCR6|IL6ST
cluster3	CD4_TCM	Longevity regulating pathway	hsa04211	3	2.67E-04	SESN3|ATG13|RHEB
cluster3	CD4_TCM	Th1 and Th2 cell differentiation	hsa04658	3	2.94E-04	HLA-DPA1|IL2RB|MAF
cluster3	CD4_TCM	Th17 cell differentiation	hsa04659	3	4.51E-04	HLA-DPA1|IL2RB|IL6ST
cluster3	CD4_TCM	Autophagy - animal	hsa04140	3	7.51E-04	ATG13|ERN1|RHEB
cluster3	CD4_TCM	Human cytomegalovirus infection	hsa05163	3	3.65E-03	RHEB|PTGER2|CCL5
cluster3	CD4_TCM	Inflammatory bowel disease (IBD)	hsa05321	2	3.73E-03	HLA-DPA1|MAF
cluster3	CD4_TCM	Rheumatoid arthritis	hsa05323	2	7.06E-03	HLA-DPA1|CCL5
cluster3	CD4_TCM	Lysosome	hsa04142	2	1.24E-02	CD63|CTSW
cluster3	CD4_TCM	Systemic lupus erythematosus	hsa05322	2	1.44E-02	HLA-DPA1|ACTN4
cluster3	CD4_TCM	Sulfur metabolism	hsa00920	1	1.47E-02	SQOR
cluster3	CD4_TCM	Apoptosis	hsa04210	2	1.50E-02	ERN1|CTSW
cluster3	CD4_TCM	Measles	hsa05162	2	1.54E-02	IL2RB|SLAMF1
cluster3	CD4_TCM	Ubiquinone and other terpenoid-quinone biosynthesis	hsa00130	1	1.61E-02	VKORC1
cluster3	CD4_TEM	Allograft rejection	hsa05330	4	2.14E-06	HLA-DPA1|FAS|HLA-DRB1|HLA-DPB1
cluster3	CD4_TEM	Influenza A	hsa05164	6	2.18E-06	HLA-DPB1|CASP1|FAS|HLA-DRB1|OAS2|HLA-DPA1
cluster3	CD4_TEM	Graft-versus-host disease	hsa05332	4	2.83E-06	HLA-DPA1|FAS|HLA-DRB1|HLA-DPB1
cluster3	CD4_TEM	Type I diabetes mellitus	hsa04940	4	3.38E-06	HLA-DPA1|FAS|HLA-DRB1|HLA-DPB1
cluster3	CD4_TEM	Th17 cell differentiation	hsa04659	5	4.68E-06	HLA-DPA1|HLA-DPB1|HLA-DRB1|GATA3|IL6ST
cluster3	CD4_TEM	Autoimmune thyroid disease	hsa05320	4	7.35E-06	HLA-DPA1|FAS|HLA-DRB1|HLA-DPB1
cluster3	CD4_TEM	Inflammatory bowel disease (IBD)	hsa05321	4	1.58E-05	HLA-DPA1|HLA-DPB1|HLA-DRB1|GATA3
cluster3	CD4_TEM	Antigen processing and presentation	hsa04612	4	2.98E-05	HLA-DPA1|HLA-DPB1|HLA-DRB1|CD74
cluster3	CD4_TEM	Alzheimer disease	hsa05010	5	4.16E-05	FAS|UQCRC1|CAPN2|NDUFA8|NCSTN
cluster3	CD4_TEM	Asthma	hsa05310	3	5.55E-05	HLA-DPA1|HLA-DPB1|HLA-DRB1

cluster3	CD4_TEM	Th1 and Th2 cell differentiation	hsa04658	4	5.80E-05	HLA-DPA1|HLA-DPB1|HLA-DRB1|GATA3
cluster3	CD4_TEM	Epstein-Barr virus infection	hsa05169	5	8.77E-05	HLA-DPA1|FAS|HLA-DRB1|OAS2|HLA-DPB1
cluster3	CD4_TEM	Intestinal immune network for IgA production	hsa04672	3	1.99E-04	HLA-DPA1|HLA-DPB1|HLA-DRB1
cluster3	CD4_TEM	Systemic lupus erythematosus	hsa05322	4	2.29E-04	HLA-DPA1|HLA-DPB1|HLA-DRB1|ACTN4
cluster3	CD4_TEM	Viral myocarditis	hsa05416	3	3.52E-04	HLA-DPA1|HLA-DPB1|HLA-DRB1
cluster9	CD8_TEM	Oxidative phosphorylation	hsa00190	3	2.67E-04	MT-ND1|MT-ND2|MT-ND4
cluster9	CD8_TEM	Parkinson disease	hsa05012	3	3.22E-04	MT-ND1|MT-ND2|MT-ND4
cluster9	CD8_TEM	Retrograde endocannabinoid signaling	hsa04723	3	3.62E-04	MT-ND1|MT-ND2|MT-ND4
cluster9	CD8_TEM	Thermogenesis	hsa04714	3	1.29E-03	MT-ND1|MT-ND2|MT-ND4
cluster9	CD8_TEM	Cytokine-cytokine receptor interaction	hsa04060	3	2.54E-03	IL7R|LTB|CX3CR1
cluster9	CD8_TEM	Natural killer cell mediated cytotoxicity	hsa04650	2	6.65E-03	KLRK1|FCGR3A
cluster9	CD8_TEM	Circadian rhythm	hsa04710	1	2.89E-02	PER1
cluster9	CD8_TEM	Autophagy - other	hsa04136	1	2.98E-02	ATG2A
cluster9	CD8_TEM	Primary immunodeficiency	hsa05340	1	3.43E-02	IL7R
cluster9	CD8_TEM	Vasopressin-regulated water reabsorption	hsa04962	1	4.04E-02	AQP3
cluster9	CD8_TEM	Metabolic pathways	hsa01100	4	4.15E-02	MT-ND1|PDE4B|MT-ND4|MT-ND2
cluster9	CD8_TEM	Malaria	hsa05144	1	4.48E-02	KLRK1
cluster9	CD8_TEM	Staphylococcus aureus infection	hsa05150	1	6.14E-02	FCGR3A
cluster9	CD8_TEM	Central carbon metabolism in cancer	hsa05230	1	6.22E-02	SLC7A5
cluster9	CD8_TEM	Leishmaniasis	hsa05140	1	6.65E-02	FCGR3A
